# Personalized intervention cardiology with transcatheter aortic valve replacement made possible with a non-invasive monitoring and diagnostic framework

**DOI:** 10.1038/s41598-021-85500-2

**Published:** 2021-05-25

**Authors:** Seyedvahid Khodaei, Alison Henstock, Reza Sadeghi, Stephanie Sellers, Philipp Blanke, Jonathon Leipsic, Ali Emadi, Zahra Keshavarz-Motamed

**Affiliations:** 1 Department of Mechanical Engineering, McMaster University, Hamilton, ON L8S 4L7 Canada; 2 St. Paul’s Hospital, Vancouver, BC Canada; 3 Department of Radiology, University of British Columbia, Vancouver, BC Canada; 4 Department of Electrical and Computer Engineering, McMaster University, Hamilton, ON Canada; 5 School of Biomedical Engineering, McMaster University, Hamilton, ON Canada; 6 School of Computational Science and Engineering, McMaster University, Hamilton, ON Canada

**Keywords:** Engineering, Biomedical engineering

## Abstract

One of the most common acute and chronic cardiovascular disease conditions is aortic stenosis, a disease in which the aortic valve is damaged and can no longer function properly. Moreover, aortic stenosis commonly exists in combination with other conditions causing so many patients suffer from the most general and fundamentally challenging condition: complex valvular, ventricular and vascular disease (C3VD). Transcatheter aortic valve replacement (TAVR) is a new less invasive intervention and is a growing alternative for patients with aortic stenosis. Although blood flow quantification is critical for accurate and early diagnosis of C3VD in both pre and post-TAVR, proper diagnostic methods are still lacking because the fluid-dynamics methods that can be used as engines of new diagnostic tools are not well developed yet. Despite remarkable advances in medical imaging, imaging on its own is not enough to quantify the blood flow effectively. Moreover, understanding of C3VD in both pre and post-TAVR and its progression has been hindered by the absence of a proper non-invasive tool for the assessment of the cardiovascular function. To enable the development of new non-invasive diagnostic methods, we developed an innovative image-based patient-specific computational fluid dynamics framework for patients with C3VD who undergo TAVR to quantify metrics of: (1) global circulatory function; (2) global cardiac function as well as (3) local cardiac fluid dynamics. This framework is based on an innovative non-invasive Doppler-based patient-specific lumped-parameter algorithm and a 3-D strongly-coupled fluid-solid interaction. We validated the framework against clinical cardiac catheterization and Doppler echocardiographic measurements and demonstrated its diagnostic utility by providing novel analyses and interpretations of clinical data in eleven C3VD patients in pre and post-TAVR status. Our findings position this framework as a promising new non-invasive diagnostic tool that can provide blood flow metrics while posing no risk to the patient. The diagnostic information, that the framework can provide, is vitally needed to improve clinical outcomes, to assess patient risk and to plan treatment.

## Introduction

One of the most common acute and chronic cardiovascular disease conditions is aortic stenosis, a disease in which the aortic valve is damaged and can no longer function properly. This condition can progress to heart failure through the rapid deterioration of the pumping action of the heart. Heart failure is a disease associated with high mortality and morbidity rates that is increasing in prevalence, affecting at least 26 million people worldwide. It is responsible for about $108 billion per year, or 1–3%, of global health expenditures^1^. For aortic stenosis patients, heart failure is the primary cause of death, and half of them will die within two years of symptom onset if aortic valve disease is left untreated^2^. Prior hospitalization due to heart failure is associated with poor outcomes following aortic stenosis intervention; some research suggests that by performing an earlier treatment, before patients experience hospitalization for heart failure, outcomes may be improved^2,3^. It is important to note that aortic valve disease commonly exists in combination with other conditions, so many patients suffer from the most general and fundamentally challenging condition: complex valvular, ventricular and vascular diseases (C3VD). In C3VD, mechanical interactions occur between multiple valvular, ventricular and vascular pathologies wherein the physical phenomena associated with each pathology exhibit magnified effects on the cardiovascular system due to the presence of the other cardiovascular conditions^4–10^.

Transcatheter aortic valve replacement (TAVR) is an emerging minimally invasive intervention for patients with aortic stenosis *across a broad risk spectrum*. Prior to the recent introduction of TAVR, the only possible choice for high-risk patients with aortic stenosis was surgical replacement of the aortic valve (SAVR). TAVR is a growing alternative to surgical intervention that has provided positive outcomes and has reduced the mortality rate, with many patients experiencing a significant improvement following intervention. TAVR is also increasingly being used in lower-risk patients who may be younger and/or have moderate valvular disease. *However, there are risks associated with TAVR, because in some cases, the situation worsens or the pre-existing cardiovascular disease changes to another form of cardiovascular disease*^4–6,8^. The following series of questions must be answered before and after TAVR to ensure the procedure is completed safely and effectively: What impacts will the procedure have on the heart mechanics and function? When is the best time to perform the intervention? Is there a means to assess which patients will have a better or worse outcome? If performed, what impacts will there be on the cardiac function, circulatory mechanics and valve function? A tool that can answer these questions for each patient while considering their specific conditions is highly needed.

*"Cardiology is flow”*^11^*,* and therefore, the essential sources of cardiovascular mortality and morbidity can be explained on the basis of adverse hemodynamics: abnormal biomechanical forces and flow patterns, leading to the development and progression of cardiovascular disease^12^. *Despite its importance, there exists no diagnostic tool that can quantify fluid dynamics* for many cardiovascular diseases, including C3VD and TAVR, in a patient-specific manner, because the fluid-dynamics methods that can be used as engines of new diagnostic tools are not yet well developed. Moreover, there are varying prognostic implications, so careful diagnosis is vital^13^. In this research, we contributed to advancing computational mechanics as a powerful means to enhance clinical measurements and medical imaging to make novel diagnostic methods for patients with C3VD and TAVR *that pose no risk to the patient.*

The heart resides in a sophisticated vascular network whose loads impose boundary conditions on the heart function. Precise and effective diagnosis hinges on the quantification of the following three Requirements: **global** hemodynamics: (1)*Metrics of circulatory function*, e.g., detailed information of the dynamics of the circulatory system, and (2) *Metrics of cardiac function*, e.g., heart workload and the breakdown of workload contributions from each cardiovascular disease component, and of the **local** hemodynamics: (3) *Cardiac fluid dynamics*, e.g., details of the instantaneous 3-D flow, vortex formation, growth, eventual shedding, and their effects on fluid transport and stirring inside the heart. Despite its importance and advances in medical imaging, as described in the following, the current clinical diagnostic tools cannot sufficiently quantify flow conditions in patients with many cardiovascular diseases, including in patient with C3VD who undergo TAVR. Cardiac catheterization, a clinical gold standard, evaluates heart function metrics. However, it is invasive and carries high risk^14^, and is not practical for diagnosis. Phase-contrast magnetic resonance imaging (MRI) can provide velocity field. but it has a lower temporal resolution than Doppler echocardiography (DE) resolution^15,16^. It is important to note that MRI cannot be used for patients with most implanted medical devices except safely for MRI-conditional devices. 2-D Doppler echocardiography is risk-free and the most practical tool for hemodynamics and has high temporal resolution (unlike 3-D DE that suffers from low temporal resolution). Studies with 4-D phase-contrast magnetic resonance unveiled that the intraventricular flow is mainly parallel to the apical long-axis plane, and measurement of 2-D flow on this plane can provide a very good estimate of the 3-D flow^17^. This makes the apical long-axis plane, passing through the left ventricle (LV) apex and the centers of the mitral valve, aortic valve, left atrium and proximal ascending aorta, the optimal 2-D representation of the 3-D LV flow. Recent advances in DE to measure flow velocity are: (1) Echo-PIV, is a promising adaptation of Particle Image Velocimetry (PIV)^18–20^, but it may underestimate high velocities^21^ so it may hinder appropriate diagnosis^22^; (2) Colour-Doppler vector flow mapping (VFM) can calculate the flow velocity using color DE images^23^; color DE cannot measure flow velocity in the direction vertical to the beam. Despite all the potential that DE has and the progress made with VFM and Echo-PIV, there is no DE method that can comprehensively evaluate local hemodynamics in the LV, valves, ascending aorta and left atrium in terms of vortical structures, their temporal evolutions, fluid transport and mixing. There is also no DE method to evaluate global hemodynamics and to break down the contributions of each cardiovascular system component.

In this study, we developed a highly innovative computational-mechanics framework that can eventually, upon further development and validation, function as a diagnostic tool for the most general and fundamentally challenging condition, C3VD, in both pre and post TAVR states. Such a diagnostic tool should dynamically couple the local hemodynamics with the global circulatory cardiovascular system to provide a framework to evaluate the effects of the global (Requirements #1 and #2) and local hemodynamics (Requirement #3) in a patient-specific manner. For this purpose, we developed a framework based on an innovative Doppler-based patient-specific lumped-parameter algorithm and a 3-D strongly-coupled fluid–solid interaction. It satisfies all three requirements for developing a clinically-effective computational diagnostic framework that can quantify local and global hemodynamics in patients who have C3VD in both pre and post intervention states. Our lumped-parameter algorithm allows for the analysis of any combination of complex valvular, vascular and ventricular diseases in C3VD patients in both pre and post intervention states by *purposefully using limited and reliable non-invasive input parameters acquired with Doppler echocardiography and sphygmomanometers to continuously calculate patient-specific global hemodynamics quantities* (Requirements #1 and #2)*.* We used the clinical data of eleven patients with C3VD in both pre and post TAVR conditions (twenty-two cases) not only to validate the proposed framework but also to demonstrate its diagnostic abilities by providing novel analyses and interpretations of clinical data. The validation was done against clinical cardiac catheterization data^24^ and clinical Doppler echocardiographic measurements.

## Methods

**Figure 1:**
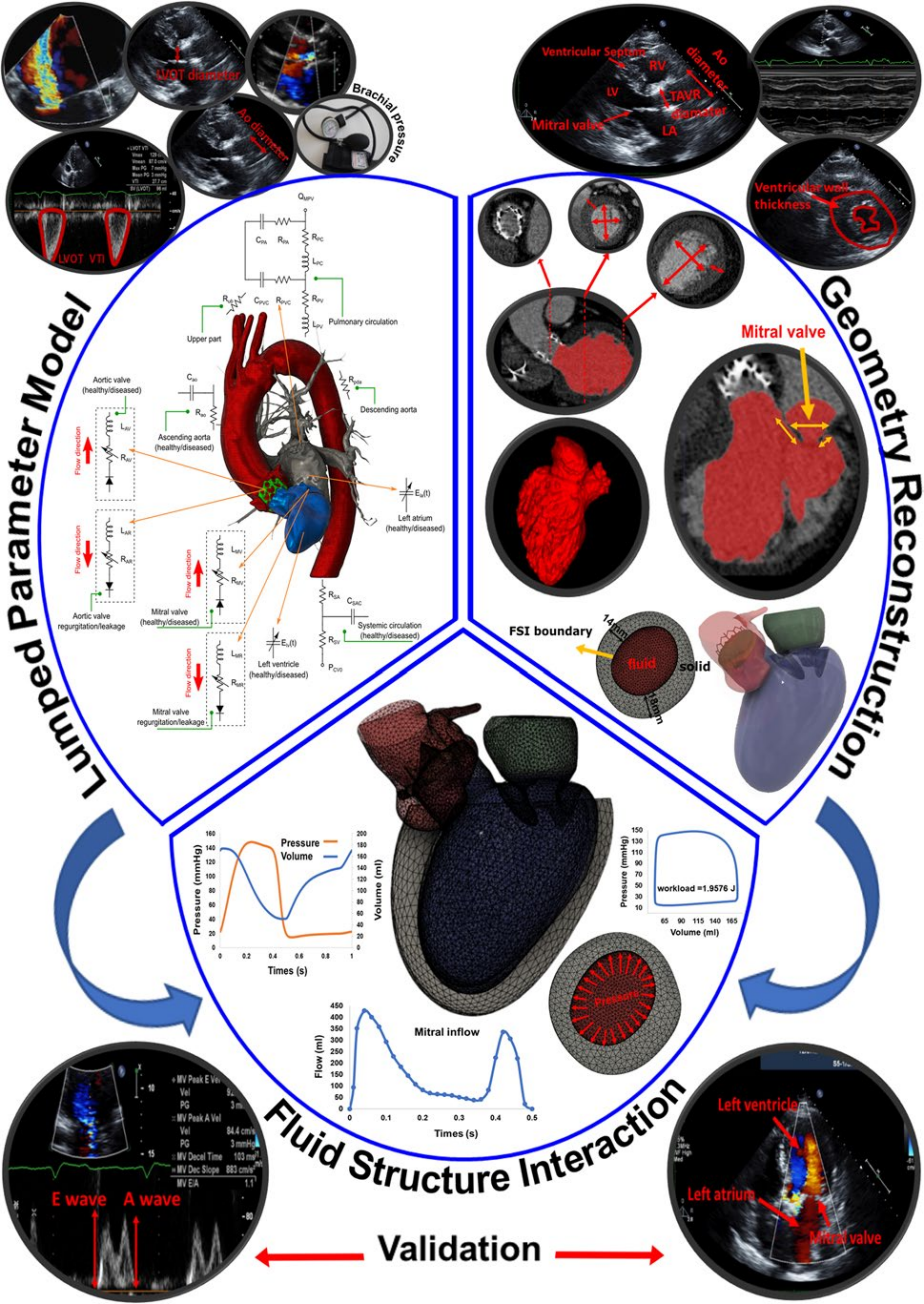
*Schematic diagram of computational domain*. Anatomical and electrical schematic diagrams of the lumped parameter modeling. This model includes the following sub-models. (1) left atrium, (2) left ventricle, (3) aortic valve, (4) mitral valve, (5) systemic circulation, and 6) pulmonary circulation. Abbreviations are the same as in Table 1. Input parameters were measured using Doppler echocardiography and sphygmomanometer. *Simulation domain and FSI modeling.* Imposing correct boundary conditions to the flow model is critical because the local flow dynamics are influenced by downstream and upstream conditions. Patient-specific LPM simulating the function of the left side of the heart was coupled to the inlet of the mitral valve model. This data was obtained from the patient-specific image-based lumped parameter model. Input parameters to the lumped parameter algorithm were reliable measured using OsiriX imaging software (OsiriX version 8.0.2; Pixmeo, Switzerland). We used ITK-SNAP (version 3.8.0-BETA) to segment and reconstruct the 3-D geometries of the complete ventricle using CT images. Geometries were used for investigating hemodynamics using FSI and LPM. Mesh for all models was generated using SALOME (an open-source mesh generation software).

**Figure 2:**
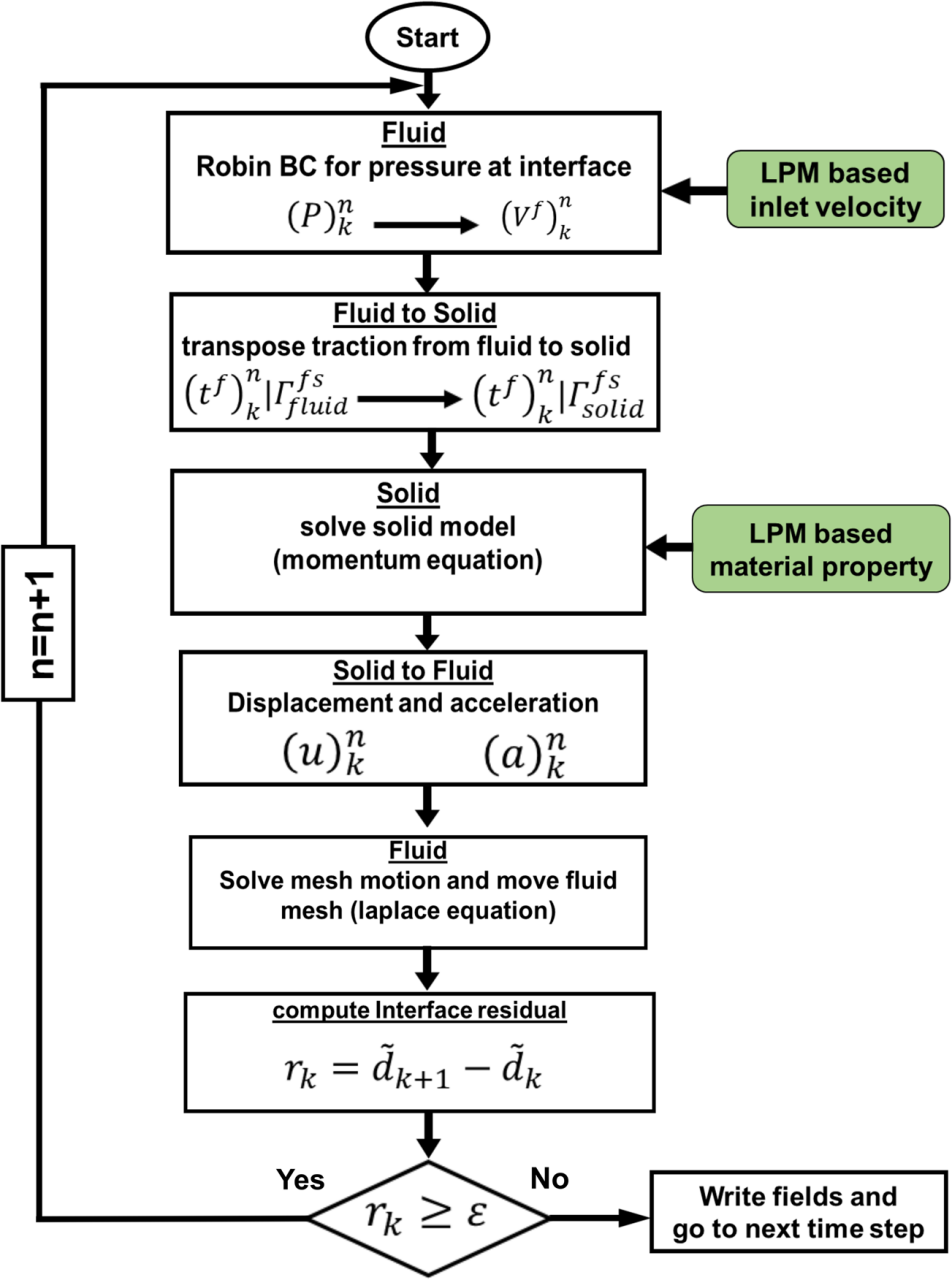
LPM and strongly coupled FSI algorithm flow chart.

**Figure 3:**
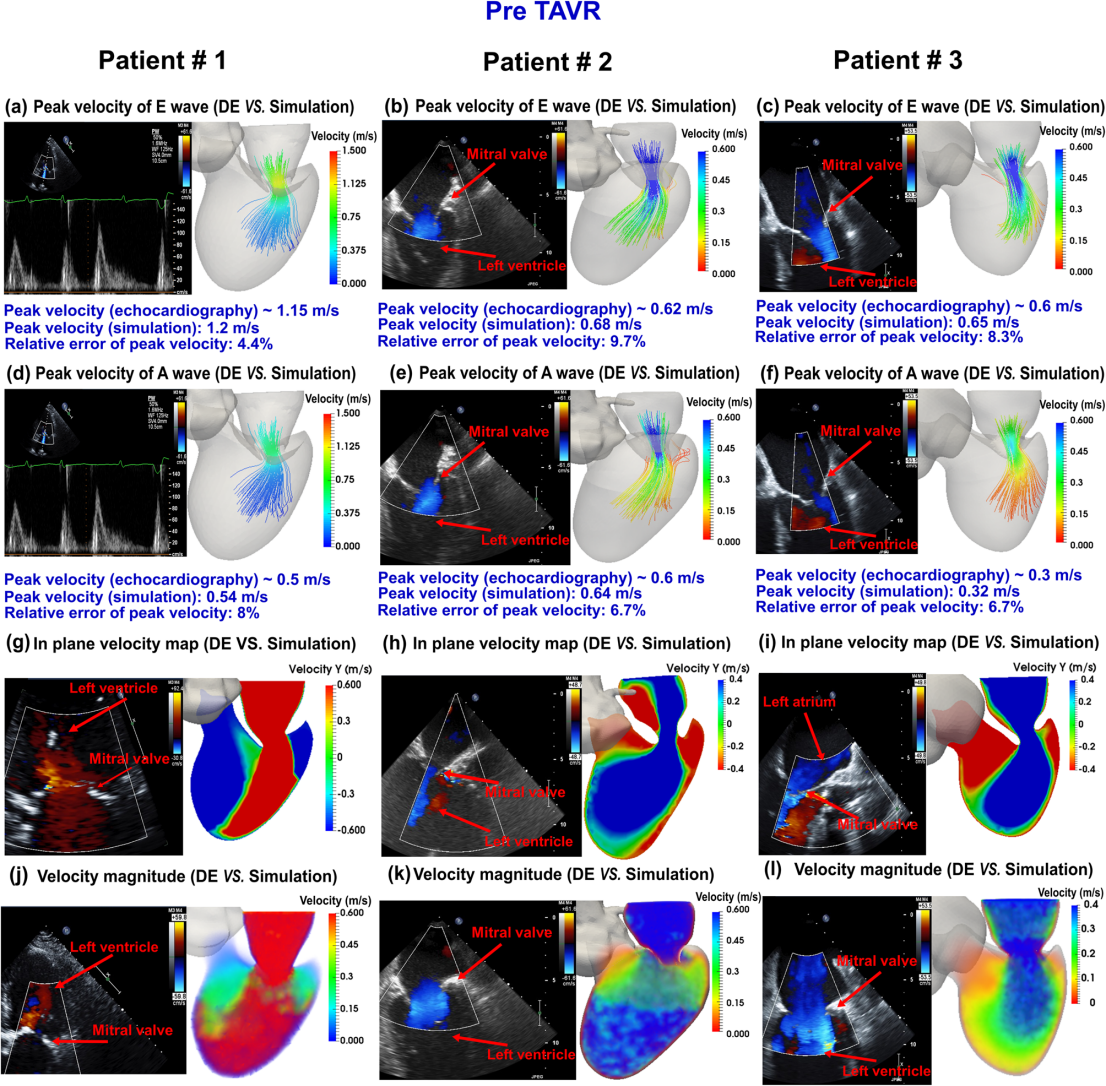
*Validation*: Doppler-based LPM and FSI framework vs. clinical Doppler echocardiography data in pre-TAVR condition. (**a**–**f**) Trans-mitral velocity during diastole in patients #1 to #3; (**g**–**i**) Left ventricle flow (apical view) during diastole in patients #1 to #3; (**j**–**l**) Trans-mitral and left ventricle flow (apical view) in patients #1 to #3.

**Figure 4:**
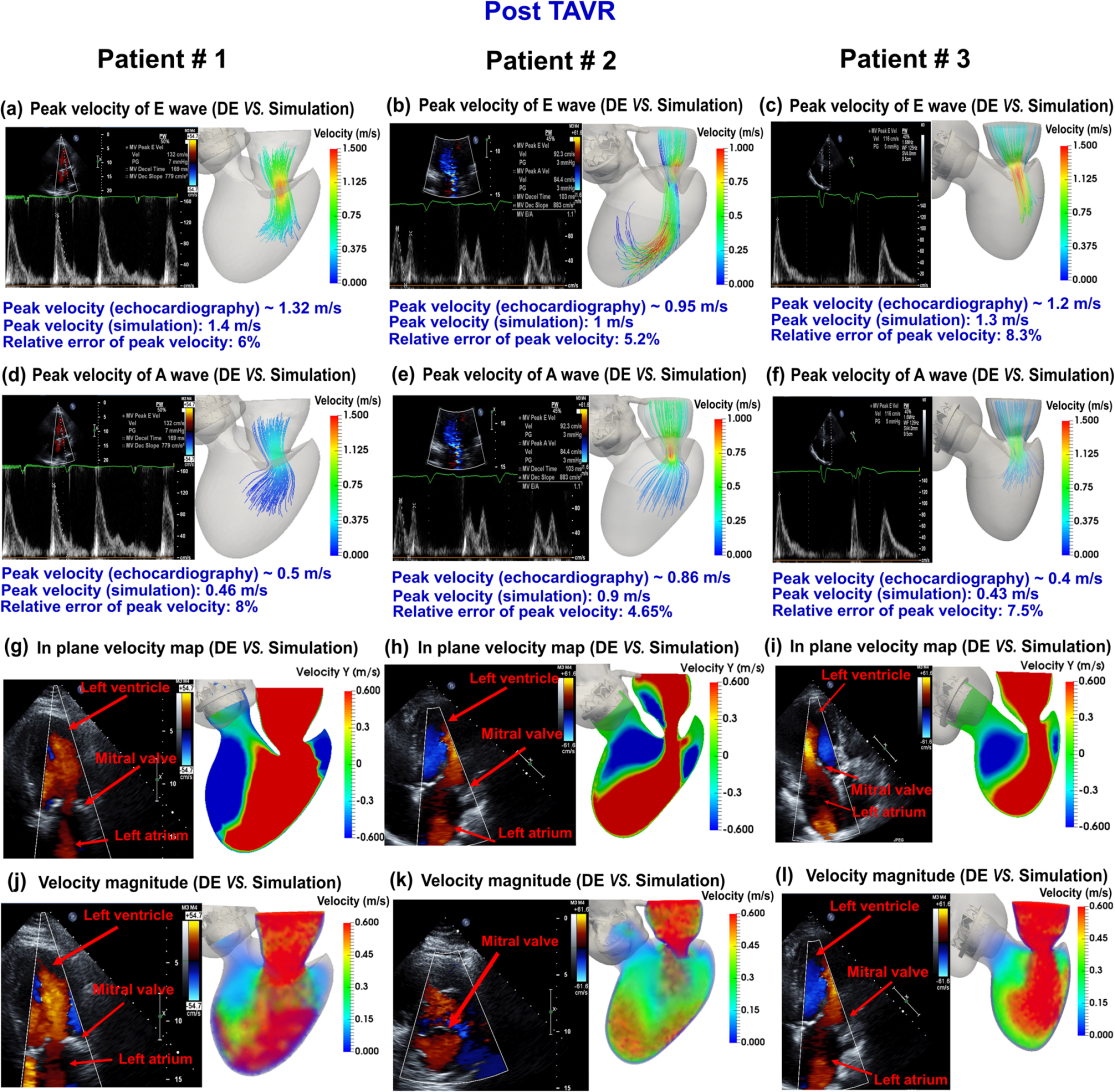
*Validation*: Doppler-based LPM and FSI framework versus clinical Doppler echocardiography data in post-TAVR condition. (**a**–**f**) Trans-mitral velocity during diastole in patients #1 to #3; (**g**–**i**) Left ventricle flow (apical view) during diastole in patients #1 to #3; (**j**, **l**) Trans-mitral and left ventricle flow (apical view) during diastole in patients #1 and #3; (**k**) Mitral valve flow (parasternal short axis view) in patient #2.

We developed an innovative image-based computational fluid dynamics framework to quantify: (1) metrics of circulatory function (global hemodynamics); (2) metrics of cardiac function (global hemodynamics) as well as (3) cardiac fluid dynamics (local hemodynamics) in patients with C3VD in both pre and post TAVR states. This framework is based on an innovative non-invasive Doppler-based patient-specific lumped-parameter algorithm that allows for the analysis of any combination of complex valvular, vascular and ventricular diseases^24^, and a 3-D strongly-coupled fluid–solid interaction (FSI) (Fig. 1: schematic diagram; Fig. 2: algorithm flow chart; Table 1). Calculations of this computational fluid dynamics framework were validated against clinical cardiac catheterization data^24^ and Doppler echocardiographic measurements (Figs. 3 and 4).

**Table 1:** Cardiovascular parameters.

Description	Abbreviation	Value
**Valve parameters**		
Effective orifice area	EOA	Measured using DE
Energy loss coefficient	E_L_Co	$\frac{(EOA)A}{{A - EOA}}$
		EOA and A are measured using DE
Variable resistance	R_AV_ and R_AR_	$\frac{\rho }{{2\left. {E_{L} Co} \right|_{AV}^{2} }}Q(t)\quad \& \quad \frac{\rho }{{2\left. {E_{L} Co} \right|_{AR}^{2} }}Q(t)$
	R_MV_ and R_MR_	$\frac{\rho }{{2\left. {EOA} \right|_{MV}^{2} }}Q_{MV} (t)\quad \& \quad \frac{\rho }{{2\left. {EOA} \right|_{MR}^{2} }}Q(t)$
Inductance	L_AV_ and L_AR_	$\frac{2\pi \rho }{{\sqrt {\left. {E_{L} Co} \right|_{AV} } }}\quad \& \frac{2\pi \rho }{{\sqrt {\left. {E_{L} Co} \right|_{AR} } }}$
	L_MV_ and L_MR_	$\frac{{M_{MV} }}{{EOA_{MV} }}\quad \& \quad \frac{{M_{MV} }}{{EOA_{MR} }}$
Inertance (mitral valve)	M_MV_	Constant value: 0.53 gcm^−2^
**Systematic circulation parameters**		
Aortic resistance	R_ao_	Constant value: 0.05 mmHg s mL^−1^
Aortic compliance	C_ao_	Initial value: 0.5 mL/mmHg
		Optimized based on brachial pressures (*systolic and diastolic brachial pressures are optimization constraints*)
Systemic vein resistance	R_SV_	0.05 mmHg s mL^−1^
Systemic arteries and veins compliance	C_SAC_	Initial value: 2 mL/mmHg
		Optimized based on brachial pressures (*systolic and diastolic brachial pressures are optimization constraints*)
systemic arteries resistance (including arteries, arterioles and capillaries)	R_SA_	Initial value: 0.8 mmHg s mL^−1^
		Optimized based on brachial pressures (*Systolic and diastolic brachial pressures are optimization constraints*)
Upper body resistance	R_ub_	Adjusted to have 15% of total flow rate in healthy case^33^
Proximal descending aorta resistance	R_pda_	Constant value: 0.05 mmHg s mL^−1^
Elastance function*		
Maximum elastance	E_max_	2.1 (LV)
		0.17 (LA)
Minimum elastance	E_min_	0.06 (LV, LA)
Elastance ascending gradient	m_1_	1.32 (LV, LA)
Elastance descending gradient	m_2_	27.4 (LV)
		13.1 (LA)
Elastance ascending time translation	$\tau_{1}$	0.269 T (LV)
		0.110 T (LA)
Elastance descending time translation	$\tau_{2}$	0.452 T (LV)
		0.18 T (LA)
Elastance normalization	N	$\frac{{E_{MAX} - E_{MIN} }}{2}$
**Pulmonary circulation parameters**		
Pulmonary vein inertance	L_PV_	Constant value:0.0005 mmHg s^2^ mL^−1^
Pulmonary vein resistance	R_PV_	Constant value: 0.002 mmHg s mL^−1^
Pulmonary vein and capillary resistance	R_PVC_	Constant value: 0.001 mmHg s mL^−1^
Pulmonary vein and capillary compliance	C_PVC_	Constant value: 40 mL/mmHg
Pulmonary capillary inertance	L_PC_	Constant value: 0.0003 mmHg s^2^ mL^−1^
Pulmonary capillary resistance	R_PC_	Constant value: 0.21 mmHg s mL^−1^
Pulmonary arterial resistance	R_PA_	Constant value: 0.01 mmHg s mL^−1^
Pulmonary arterial compliance	C_PA_	Constant value: 4 mL/mmHg
Mean flow rate of pulmonary valve	Q_MPV_	*Forward LVOT-SV* is the only input flow condition (measured using DE). *Q*_*MPV*_ *is a flow parameter that was optimized so that the lump-parameter model could reproduce the desirable DE-measured Forward LVOT-SV*
**Input flow condition**		
Forward left ventricular outflow tract stroke volume	Forward LVOT-SV	Measured using DE
**Output condition**		
Central venous pressure	P_CV0_	Constant value: 4 mmHg
**Other**		
Constant blood density	$\rho$	Constant value: 1050 kg/m^3^
Heart rate	HR	Measured using DE
Duration of cardiac cycle	T	Measured using DE
Systolic end ejection time	T_EJ_	Measured using DE
End diastolic volume	EDV	Measured using DE
End systolic volume	ESV	Measured using DE

Summarized parameters used in the lumped-parameter modeling to simulate all patient-specific cases.

### Clinical medical imaging

#### Study population and data acquisition

We retrospectively selected 11 C3VD patients with severe aortic valve stenosis who underwent TAVR between 2013 and 2017 at St. Paul’s Hospital (Vancouver, Canada; N = 11). Informed consents were obtained from all human participants and the protocols were reviewed and approved by the Institutional Review Boards of the institution (the Clinical Research Ethics Board; CREB). The selections were done by operators blinded to the objectives and contents of this study. All methods and measurements were performed in accordance with relevant guidelines and regulations including guidelines of the American College of Cardiology and American Heart Association. Demographic and procedural data were collected from the patient medical records. The clinical outcome was evaluated using medical records and the New York Heart Association (NYHA) functional class, assessed at baseline and at 90-days post-TAVR. The protocol was reviewed and approved by the Ethics Committee of the institutions. Data were acquired at two time points: pre-procedure and 90-days post-procedure. Valve type and size were decided prior to the procedure by the local clinical team based on pre-procedural echocardiographic, computed tomographic, and angiographic imaging and data.

#### Doppler echocardiography (DE)

Doppler echocardiography (DE) data included raw images and documented reports that were collected at baseline and at 90-days post-procedure. Echocardiograms and reports were reviewed and analyzed in a blinded fashion by three senior cardiologists using OsiriX imaging software (OsiriX version 8.0.2; Pixmeo, Switzerland) as recommended by the American Society of Echocardiography (ASE)^25^. The following metrics were measured:

##### Input parameters of the LPM algorithm

The algorithm used the following input parameters that all can be reliably measured using DE: forward left ventricular outflow tract stroke volume, heart rate, ejection time, ascending aorta area, left ventricular outflow tract area, aortic valve effective orifice area, mitral valve effective orifice area, and grading of aortic and mitral valves regurgitation severity. These parameters were measured in the parasternal long axis, parasternal short axis, apical two-chamber, apical four-chamber and apical five-chamber views of the heart as recommended by the ASE^25^ (see Fig. 1 for details).

##### Geometrical parameters

We used the parasternal long axis, parasternal short axis, M-Mode, apical two-chamber and apical four-chamber views of the heart to measure the following parameters: height, diameter and wall thickness of the LV; and leaflet and annulus sizes of the aortic and mitral valves. Such DE-based measurements give us indispensable insights about the patients’ state, and they are extremely helpful to verify the same measurements made in reconstructed 3-D models based on CT data.

### Computed tomography (CT)

CT data included images and documented reports that were collected at baseline and at 90-days post-procedure. We used the data as follows:

#### Model reconstruction

We used CT images from patients to segment and reconstruct the 3-D geometries of the complete ventricle (ventricle, TAVR, ascending aorta, mitral valve and left atrium) employing ITK-SNAP (version 3.8.0-BETA)^26^, a 3-D image processing and model generation software package (Fig. 1). These 3-D reconstructions were used for FSI simulations.

### Lumped-parameter model

We developed an innovative non-invasive image-based patient-specific diagnostic, monitoring and predictive computational-mechanics framework for *Complex valvular-vascular-ventricular diseases (C3VD)*. For simplicity, this C3VD computational mechanics framework is called C3VD-CMF, described in details elsewhere^24^. C3VD-CMF enables the quantification of (1) details of the physiological pulsatile flow and pressures throughout the heart and circulatory system; (2) heart function metrics, e.g., left ventricle workload and instantaneous left ventricular pressure, etc. C3VD-CMF also provides the breakdown of effects that each disease constituent imposes on the global function of the cardiovascular system. Currently, none of the above metrics can be obtained noninvasively in patients, and when invasive procedures are undertaken, the collected metrics cannot be as complete as the results that C3VD-CMF provides by any means^24^.

The developed algorithm (C3VD-CMF) consists of a parameter estimation algorithm and a lumped-parameter model that includes several sub-models allowing analysis of any combination of complex valvular, vascular and ventricular diseases in both pre and post intervention conditions: (1) left atrium, (2) left ventricle, (3) aortic valve, (4) mitral valve, (5) systemic circulation and 6) pulmonary circulation (Fig. 1; Table 1). The algorithm uses the following input parameters that can all be measured reliably using Doppler echocardiography: forward left ventricular outflow tract stroke volume heart rate, ejection time, ascending aorta area, left ventricular outflow tract area, aortic valve effective orifice area and mitral valve effective orifice area. Other input parameters of the model are systolic and diastolic blood pressures measured using sphygmomanometers. The innovative lumped-parameter model calculations were validated against cardiac catheterization data in forty-nine patients with C3VD^24^. Examples of C3VD components include: valvular disease (e.g., aortic valve stenosis, mitral valve stenosis, aortic valve regurgitation and mitral valve insufficiency), ventricular disease (e.g., left ventricle dysfunction and heart failure), vascular disease (e.g., hypertension), paravalvular leaks and LV outflow tract obstruction in patients with implanted cardiovascular devices such as transcatheter valve replacement (TVR), changes due to surgical procedures for C3VD (e.g., valve replacement and left ventricular reconstructive surgery), etc.^4,6–8,27^. Some sub-models have already been used^7,27–32^ and validated against in vivo cardiac catheterization (N = 34)^33^ and in vivo MRI data (N = 57)^34^.

#### Heart-arterial model

### Left ventricle

Coupling between LV pressure and volume was achieved through a time varying elastance E(t) as follows:

1 $$E(t) = \frac{{P_{LV} (t)}}{{V(t) - V_{0} }}$$

where $P_{LV} (t)$, $V(t)$ and $V_{0}$ are LV time-varying pressure, time-varying volume and unloaded volume, respectively. To model the LV elastance finction (E(t)), we need a double Hill function as follows^24^.

2 $$E (t) = N\left( {\frac{{\left( {\frac{t}{{\tau_{1} }}} \right)^{m1} }}{{1 + \left( {\frac{t}{{\tau_{1} }}} \right)^{m1} }}} \right)\left( {\frac{1}{{1 + \left( {\frac{t}{{\tau_{2} }}} \right)^{m2} }}} \right) + E_{\min }$$

3 $$N=(Emax-Emin)/2$$

where $N$, $\tau_{1}$, $\tau_{2}$, $m_{1}$, $m_{2}$, Emax and Emin are elastane normalization, ascending time translation, descending time translation, ascending gradient, descending gradient, maximum elastance and minimum elastance, respectively (see Table 1). A double Hill function was believed necessary to model the contraction and relaxation in the heart chambers: in Eq. (2), the first term in brackets corresponds to the contraction of the chamber, and the second term in brackets corresponds to the relaxation of the chamber. $\tau_{1}$ and $\tau_{2}$ govern the time translation, while $m_{1}$ and $m_{2}$ govern the gradient of the elastance function. Parameter values used for the elastance function were adapted to obtain physiological waveforms for pressure, volume and flow that can be found in Table 1^1^.

### Left atrium

Coupling between LA pressure and volume was done through a time varying elastance E(t), a measure of cardiac muscle stiffness, using the same procedure as outlined above for the LV. The elastance function used for the LA is defined in Eqs. (2) and (3)^24^; parameter values used can be found in Table 1.

### Modeling heart valves

#### Modeling the aortic valve

##### Aortic valve

The aortic valve was modeled using the net pressure gradient formulation $(PG_{net} )$ across the aortic valve during LV ejection. This formulation expresses the instantaneous net pressure gradient across the aortic valve as a function of the instantaneous flow rate and the energy loss coefficient. It also links the LV pressure to the ascending aorta pressure:

4 $$\left. {PG_{net} } \right|_{AV} = \frac{2\pi \rho }{{\sqrt {\left. {E_{L} Co} \right|_{AV} } }}\frac{\partial Q(t)}{{\partial t}} + \frac{\rho }{{2\left. {E_{L} Co} \right|_{AV}^{2} }}Q^{2} (t)$$

and

5 $$\left. {E_{L} Co} \right|_{AV} = \frac{{(\left. {EOA} \right|_{AV} )A_{AO} }}{{A - \left. {EOA} \right|_{AV} }}$$

where $\left. {E_{L} Co} \right|_{AV}$, $\left. {EOA} \right|_{AV}$, $A_{AO}$, $\rho$ and $Q$ are the valvular energy loss coefficient, the effective orifice area, ascending aorta cross sectional area, blood density and transvalvular flow rate, respectively.

##### Aortic regurgitation

Aortic regurgitation (AR) was modeled using the same analytical formulation as aortic stenosis as follows. The AR pressure gradient is the difference between aortic pressure and LV pressure during diastole.

6A $$\left. {PG_{net} } \right|_{AR} = \frac{2\pi \rho }{{\sqrt {\left. {E_{L} Co} \right|_{AR} } }}\frac{\partial Q(t)}{{\partial t}} + \frac{\rho }{{2\left. {E_{L} Co} \right|_{AR}^{2} }}Q^{2} (t)$$

and

6B $$\left. {E_{L} Co} \right|_{AR} = \frac{{EOA_{AR} A_{LVOT} }}{{A_{LVOT} - EOA_{AR} }}$$

where $\left. {E_{L} Co} \right|_{AR}$, $EOA_{AR}$ and $A_{LVOT}$ are the regurgitation energy loss coefficient, regurgitant effective orifice area and LVOT area, respectively.

### Modeling the mitral valve

#### Mitral valve

The mitral valve (MV) was modeled using the analytical formulation for the net pressure gradient $\left. {(PG_{net} } \right|_{MV} )$ across the MV during LA ejection. This formulation expresses the instantaneous net pressure gradient across the LA and vena contracta as an unsteady incompressible inviscid flow. $\left. {PG_{net} } \right|_{MV}$ is expressed as a function of $\rho$, $Q_{MV}$, $EOA_{MV}$ and $M_{MV}$, where these quantities represent the density of the fluid, the transvalvular flow rate, effective orifice area and inertance, respectively.

7 $$\left. {PG_{net} } \right|_{MV} = \frac{{M_{MV} }}{{EOA_{MV} }}\frac{{\partial Q_{MV} \left( t \right)}}{\partial t} + \frac{\rho }{{2\left. {EOA} \right|_{MV}^{2} }}Q_{MV}^{2} \left( t \right)$$

#### Mitral regurgitation

Mitral regurgitation (MR) was modeled using Eq. (8). The MR pressure gradient is the difference between mitral pressure and left atrium pressure during systole.

8 $$\left. {PG_{net} } \right|_{MR} = \frac{{M_{MV} }}{{EOA_{MR} }}\frac{\partial Q(t)}{{\partial t}} + \frac{\rho }{{2\left. {EOA} \right|_{MR}^{2} }}Q^{2} (t)$$

where $\left. {EOA} \right|_{MR}$ is the MR effective orifice area.

### Pulmonary flow

The pulmonary valve flow waveform was simulated by a rectified sine curve with duration $t_{ee}$ and amplitude Q_MPV_ as follows.

9 $$Q_{PV} \left( t \right) = Q_{MPV} \sin \left( {\frac{\pi t}{{t_{ee} }}} \right),\quad {\text{t}} \le {\text{t}}_{{{\text{ee}}}} ; Q_{PV} \left( t \right) = 0,\quad {\text{t}}_{{{\text{ee}}}} < {\text{t}} \le {\text{T}}$$

where Q_MPV_, t_ee_ and T are the mean flow rate of the pulmonary valve, end-ejection time and cardiac cycle time period, respectively. In this study, it is very important to note that *forward left ventricular outflow tract stroke volume* (*Forward LVOT-SV*) is the only input flow condition that can be reliably measured using DE. Q_MPV_, the mean flow rate of the pulmonary valve, was optimized so that the lumped-parameter model could reproduce the desirable DE-measured *Forward LVOT-SV*.

#### Determining arterial compliance and peripheral resistance

The total systemic resistance was computed as the quotient of the average brachial pressure and the cardiac output (assuming a negligible peripheral venous pressure (mean ~ 5 mmHg) compared to aortic pressure (mean ~ 100 mmHg)). This total systemic resistance represents the equivalent electrical resistance for all resistances in the current model. Because the LV faces the total systemic resistance as opposed to the individual resistances, we considered the aortic resistance, $R_{ao}$, and systemic vein resistance, $R_{SV}$, as constants and adjusted the systemic artery resistance,$R_{SA}$, according to the acquired total systemic resistance. Systemic artery resistance was assessed using an optimization scheme outlined in the patient-specific parameter estimation section.

For each degree of hypertension, we fit the predicted pulse pressure to the actual pulse pressure (measured by arm cuff sphygmomanometer) obtained from clinical study by adjusting the compliances (aorta (C_ao_) and systemic (C_SAC_)). Therefore, for each degree of arterial hypertension, the compliance was evaluated using an optimization scheme outlined in the patient-specific parameter estimation section ^24^.

#### Patient-specific parameter estimation

The lumped-parameter model took the following patient-specific parameters as its inputs: forward left ventricular outflow tract stroke volume (*Forward LVOT-SV*), cardiac cycle time (T), ejection time (T_EJ_), EOA_AV_, EOA_MV_, A_AO_, A_LVOT_, EOA_AR_, EOA_MR_ and brachial systolic and diastolic pressures measured by a sphygmomanometer. The following procedure was used to set up the patient-specific lumped-parameter model:

1. *Flow inputs* The lumped-parameter model used only one reliably measured flow parameter as an input: *Forward LVOT-SV* (Eq. 10). *Forward LVOT-SV* is defined as the volume of blood that passes through the LVOT every time the heart beats.
  10 $${\text{Forward LVOT - SV}} = A_{LVOT} \times VTI_{LVOT} = \frac{{\pi \times (D_{LVOT} )^{2} }}{4} \times VTI_{LVOT}$$
  where $D_{LVOT}$, $A_{LVOT}$, and $VTI_{LVOT}$ are the LVOT diameter, LVOT area, and LVOT velocity–time integral, respectively.
2. *Time inputs* Cardiac cycle time (T) and ejection time (T_EJ_) were measured using Doppler echocardiography.
3. *Aortic valve inputs* $A_{AO}$ and $\left. {EOA} \right|_{AV}$ were calculated using Eqs. (11) and (12), respectively.
  11 $${\text{A}}_{{{\text{AO}}}} = \frac{{\pi \times (D_{AO} )^{2} }}{4}$$
  12 $$\left. {EOA} \right|_{AV} = \frac{{\text{Forward LVOT - SV}}}{{VTI_{AO} }}$$
  where $D_{AO}$ and $VTI_{AO}$ are the diameter of the ascending aorta and the velocity–time integral in the ascending aorta, respectively. To model the blood flow in the forward direction, $A_{AO}$ and $\left. {EOA} \right|_{AV}$ were then substituted into Eq. (5). Subsequently, Eq. (4) was used to calculate the constant inductance ($\frac{2\pi \rho }{{\sqrt {\left. {E_{L} Co} \right|_{AV} } }}$) and variable resistance ($\frac{\rho }{{2\left. {E_{L} Co} \right|_{AV}^{2} }}Q(t)$) parameters.
4. *Aortic regurgitation inputs* To model blood flow in the reverse direction (aortic valve insufficiency), $EOA_{AR}$ and $A_{LVOT}$ were substituted into Eq. 6 to calculate the variable resistance ($\frac{\rho }{{2\left. {E_{L} Co} \right|_{AR}^{2} }}Q(t)$) and constant inductance ($\frac{2\pi \rho }{{\sqrt {\left. {E_{L} Co} \right|_{AR} } }}$) parameters. For patients with no insufficiency, the reverse branch was not included. $A_{LVOT}$ was quantified using Doppler echocardiography measurements.
5. *Mitral valve inputs* To model the blood flow in the forward direction, the mitral valve area was substituted into Eq. (7) to calculate the constant inductance ($\frac{{M_{MV} }}{{EOA_{MV} }}$) and variable resistance ($\frac{\rho }{{2\left. {EOA} \right|_{MV}^{2} }}Q_{MV} (t)$) parameters. The mitral valve is approximately an ellipse, and its area was quantified using ${\text{A}}_{{{\text{MV}}}} \, = \,\frac{{\pi {*}d_{1} *d_{2} }}{4}$, where d_1_ and d_2_ are mitral valve diameters measured in the apical two-chamber and apical four-chamber views, respectively.
6. *Mitral regurgitation inputs* To model blood flow in the reverse direction (mitral valve insufficiency), EOA_MR_ was substituted into Eq. 8 to calculate the variable resistance ($\frac{\rho }{{2\left. {EOA} \right|_{MR}^{2} }}Q(t)$) and constant inductance ($\frac{{M_{MV} }}{{EOA_{MR} }}$) parameters. For patients with no insufficiency, the reverse branch was not included.
7. *End systolic volume and end diastolic volume* The end systolic volume (ESV) or end diastolic volume (EDV) measured using Doppler echocardiography were fed to the lumped-parameter model to adjust the starting and ending volumes in the P–V loop diagram.
8. *Left ventricle inputs* The cardiac cycle time (T) was substituted into $\tau_{1}$, $\tau_{2}$, $m_{1}$ and $m_{2}$ in Table 1, and then those values were substituted into Eq. (2) to determine the elastance function.
9. *Left atrium inputs* The cardiac cycle time (T) was substituted into $\tau_{1}$, $\tau_{2}$, $m_{1}$ and $m_{2}$ in Table 1, and then those values were substituted into Eq. (2) to determine the elastance function.

(10) *Parameter estimation for systemic circulation* Parameters R_SA_, C_SAC_ and C_ao_ were optimized so that the aortic pressure calculated using the model matched the patient’s systolic and diastolic brachial pressures measured using a sphygmomanometer (see computational algorithm section for details). The initial values of these parameters are given in Table 1.
(11) *Simulation execution* Please see the computational algorithm section.

#### Computational algorithm

The lumped-parameter model was analyzed numerically by forming and solving a system of ordinary differential equations in Matlab Simscape (MathWorks, Inc.), augmented by including additional functions written in Matlab and Simscape. Matlab’s ode23t trapezoidal rule variable-step solver was used to solve the system of differential equations with an initial time step of 0.1 ms. The convergence residual criterion was set to 10^–6^. Initial voltages and currents of the capacitors and inductors were set to zero. The model was run for several cycles (~ 150 cycles) to reach steady state before starting the response optimization process described below.

A double Hill function representation of a normalized elastance curve for human adults^35,36^ was used to generate a signal to model LV elastance. It was shown that this elastance formulation can correctly represent the LV function independent of its healthy and/or pathological condition. Simulations began at the onset of isovolumic contraction. The instantaneous LV volume, V(t), was computed using the LV pressure, P_LV_, and the time-varying elastance (Eq. 1). The LV flow rate was subsequently calculated as the time derivative of the instantaneous LV volume. The same approach was used to obtain the left atrium volume, pressure and flow rate. P_LV_ was first calculated using the initial values of the model input parameters from Table 1. The *Forward LVOT-SV* calculated using the lumped-parameter model was then fitted to the one measured (Eq. 10) by optimizing Q_MPV_ (as detailed below). Lastly, for each patient, R_SA_, C_SAC_ and C_ao_ were optimized to fit the aortic pressure from the model to the patient systolic and diastolic pressures measured using a sphygmomanometer.

#### Patient-specific response optimization

The Simulink Design Optimization toolbox was used to optimize the response of the lumped-parameter model using the trust region reflective algorithm implemented in the Matlab fmincon function. The response optimization was performed in two sequential steps with tolerances of 10^–6^. In the first step, Q_MPV_, the mean flow rate of the pulmonary valve, was optimized to minimize the error between the *Forward LVOT-SV* calculated by the lumped-parameter model and the one measured in each patient. In the second step, R_SA_, C_SAC_ and C_ao_ were optimized so that the maximum and minimum values of the aortic pressure were respectively equal to the systolic and diastolic pressures measured using a sphygmomanometer in each patient.

### Fluid–solid interaction simulation study

In this study, blood flow simulations rely on 3-D fluid–solid interaction (FSI) computational fluid dynamics using FOAM-Extend^37^ in which the system of equations governing the FSI problem is formulated using the finite volume method.

#### Governing equations for the fluid domain

Blood flow was governed by the 3D incompressible Navier–Stokes equations^38,39^ and was assumed to be a Newtonian and incompressible fluid with a dynamic viscosity of 0.004 Pa·s and a density of 1060 kg/m^3^^40^. The following continuity and momentum equations were used^41^:

13 $$\oint_{s} {(n \cdot V)ds = 0}$$

14 $$\int_{\forall } {\frac{\partial V}{{\partial t}}} d\forall + \oint_{s} {V[n \cdot V]ds = \frac{1}{\rho }} \oint_{s} {n \cdot [} \mu \nabla V]ds - \frac{1}{\rho }\int_{\forall } {\nabla pd\forall }$$

where *n* is the normal vector to the surface *S,* $\forall$ is the volume, *V* is the fluid velocity*, µ* is the fluid dynamic viscosity, *P* is the blood pressure and *ρ* is the fluid density. Due to the deformation of the fluid–solid interface, momentum Eq. (14) was deemed in the Arbitrary Lagrangian–Eulerian (ALE) form as follows^41^:

15 $$\int_{\forall } {\frac{\partial V}{{\partial t}}} d\forall + \oint_{s} {n \cdot (V - V_{s} )Vds = \frac{1}{\rho }} \oint_{s} {n \cdot [} \mu \nabla V]ds - \frac{1}{\rho }\int_{\forall } {\nabla pd\forall }$$

16 $$\frac{d}{dt}\int_{\forall } {d\forall = \oint_{s} {n \cdot V_{S} } } ds$$

where *V*_*s*_ is the velocity of the surface. The relationship between the rates of change of the cell volume and the mesh motion flux was governed by conservation law^42^. Equation 16 indicates that the rates of change of the volume and the velocity of the surface are in equilibrium^42^.

#### Governing equations for the solid domain

Because the LV is passive during diastole, its deformation depends on the tissue structure and the blood pressure inside the LV^43^. The endocardium, myocardium and epicardium are the three main layers that compose the wall of the heart. As the myocardium is located between the endocardium and epicardium and constitutes the majority of LV tissue thickness, it is primarily responsible for the mechanical behaviour of the LV wall^44^. Several empirical models have been designed to describe the passive behaviour of the myocardial layer^45–48^, of which the most notable is the Holzapfel and Ogden model^48^. Although this model has proven to be reliable, it is based on experimental results from canine or porcine hearts, with significant structural differences from human hearts^49^. Models based on animal testing are limited by the animal’s environment, morphology and physiology, which may not accurately simulate human physiology and pathophysiology in a clinical setting^50,51^. Recent studies have strived to obtain patient-specific simulations of the LV tissue by optimizing the parameters of the Holzapfel and Ogden model using displacement fields obtained from human 3-D Magnetic Resonance Imaging (MRI)^52,53^. However, in those studies, the models and the parameters were developed for non-pulsatile blood flow of healthy LVs. Additionally, the direction of myocardial fibers is required to optimize such models, thus requiring additional imaging data such as tensor diffusive MRI^54–58^. Other studies have focused on pulsatile blood flow, but they did not optimize tissue parameters to be patient-specific^59–66^. All of these studies relied on high-resolution MRI data to simulate the moving boundary of the LV wall and usually excluded the thickness of the LV in their modeling. In addition, several recent studies have combined LPM with MRI data to obtain anisotropic material properties of the LV for electro-mechanical models^55–57,67,68^. However, MRI is costly, lengthy and not possible for many patients with implanted devices like TAVR^15,16^.

In this study, we developed a method to adjust patient-specific passive material properties of the LV for patients who undergo TAVR, based on our patient-specific Doppler-based LPM algorithm^24^. *The algorithm decisively uses reliable non-invasive input parameters collected using DE. As opposed to MRI, DE is potentially the most versatile tool for hemodynamics and is low-cost and risk-free for all patients.* LV tissue was assumed to be isotropic by the Saint Venant–Kirchhoff solid model^66,69–74^. We adjusted the ventricular non-linear material properties during diastole using the results of our LPM algorithm as follows. The LPM algorithm provided the LV diastolic pressure as well as the LV pressure–volume (P–V) diagram. We applied the diastolic pressure as the boundary condition at the inner wall of the LV, and by assuming different values for material properties, we obtained a series of LV P–V diagrams. Material properties were then interpolated to find the best value that could match the LV P–V results obtained using solid modeling to those acquired using the LPM. Young’s modulus was then interpolated to match the LV P–V results to those obtained using our LPM algorithm.

According to the total Lagrangian form of the law of conservation of linear momentum, the deformation of elastic and compressible solid were considered as follows^75,76^:

17 $$\int_{{V_{0} }} {\rho_{0} } \frac{\partial }{\partial t}\left( {\frac{\partial u}{{\partial t}}} \right)dV = \oint_{{s_{0} }} {n \cdot (\sum \cdot F^{T} )ds} + \int_{{V_{0} }} {\rho_{0} bdV}$$

where the subscript 0 describes the undeformed configuration and u is the displacement vector. F is the deformation gradient tensor and can be described as *F* = *I* + *(∇u)*^*T*^*; I* is the second order identity tensor.

Also, in Eq. (17), Ʃ is the second Piola–Kirchhoff stress tensor and is described through the Cauchy stress tensor (σ) as follows:

18 $$\sigma = \frac{1}{\det F}F \cdot \Sigma \cdot F^{T}$$

Using the St. Venant–Kirchhoff constitutive material model, Ʃ is explained through isotropic Hooke’s law:

19 $$\Sigma = \lambda tr(E)I + 2\mu E$$

where µ and λ are the Lame’s constants (related to the Young’s modulus and Poisson’s ratio of material). E is the Green-Lagrangian strain tensor and is defined as follows:

20 $$E = \frac{1}{2}[\nabla u + (\nabla u)^{T} + \nabla u \cdot (\nabla u)^{T} ]$$

By substituting Eqs. (19) and (20) into Eq. (17), the governing equation for the St. Venant–Kirchhoff hyperelastic solid in the total Lagrangian form can be obtained as follows^76^:

21 $$\begin{aligned} & \int_{{V_{0} }} {\rho_{0} } \frac{\partial }{\partial t}\left(\frac{\partial u}{{\partial t}}\right)dV - \oint_{{s_{0} }} {n \cdot (2\mu + \lambda )\nabla uds} = \rho_{0} \int_{{V_{0} }} {bdV} \\ &\quad +\oint_{{s_{0} }} {n \cdot [\mu (\nabla u)^{T} } + \lambda tr(\nabla u)I - (\mu + \lambda )\nabla u + \mu \nabla u \cdot (\nabla u)^{T} + \frac{1}{2}\lambda tr[\nabla u \cdot (\nabla u)^{T} ]I + \sum \cdot \nabla u]ds \\ \end{aligned}$$

#### Fluid–structure interaction (FSI)

The fluid and solid solvers were strongly coupled together to simulate the LV under pathophysiological flow and pressure conditions. We used the partitioned approach to separately solve the system of equations of the fluid and solid domains while the data were transferred at the interface. Both the solid and fluid were modeled using a finite-volume approach to reduce the cost of transferring information between the domains^77^. The fluid and solid solvers were coupled by the kinematic and dynamic conditions for the LV. To satisfy the kinematic condition, the velocity and displacement must be continuous across the interface^76^.

22 $$u_{f,i} = u_{s,i}$$

23 $$V_{f,i} = V_{s,i}$$

where subscripts *i, s* and *f* indicate the interface, solid and fluid regions, respectively. To satisfy the dynamic condition, the forces at the interface must be in equilibrium:

24 $$n_{i} .\sigma_{f,i} = n_{i} .\sigma_{s,i}$$

The Dirichlet–Neumann procedure at the interface indicates that the fluid domain is solved for a given velocity/displacement while the solid domain is solved for a given traction^76^.

#### Grid study

Mesh for all models was generated using SALOME, an open-source mesh generation software^78^. Spatial mesh resolution had been examined to optimize the number of elements for FSI simulation. Mesh definition (with optimized non-orthogonality and skewness values) for both fluid and solid domains was considered acceptable when no significant difference (less than 2%) between successive meshes was noticed in velocity profiles. To maintain the initial quality of the cells, the fluid dynamic mesh was governed by Laplace mesh motion, which was controlled by variable diffusivity^41,79^. Mesh at the interface of the fluid and solid domains was not conformal, and consequently, interpolation was performed between the fluid and solid boundaries. The interpolation was performed based on the face-interpolation and vertex-interpolation procedures^41^. Moreover, time step independency had been studied for all models. The solution marched in time with a time step of 0.0001 s, yielding a maximum Courant number of 0.2. To improve the accuracy of the numerical simulation and to reduce numerical dispersion, the Courant number was lower than 0.25 for all simulations investigated in this study. Convergence was obtained when all residuals reached a value lower than 10^–6^. Temporal discretization was performed with a second-order Euler backward scheme, and a second-order accurate scheme was used for the spatial discretization.

#### Boundary conditions and material properties

Imposing the correct boundary conditions to the flow model is critical because the local flow dynamics are influenced by downstream and upstream conditions. Boundary conditions were obtained from our patient-specific image-based lumped-parameter algorithm (Fig. 1)^24^: (1) to provide the time-dependent trans-mitral blood flow rate with the physiologic E and A waveforms; (2) to calculate material properties; (3) to provide the reference pressure, set inside the LV. All geometries were reconstructed based on images obtained at the beginning of diastole and, because LV diastolic dysfunction occurs in the left ventricular filling phase, all simulations were performed during diastole. Therefore, the TAVR was modeled to be rigidly closed and the mitral valve was modeled fully open during the diastolic phase. The effect of the chordae tendineae was not considered as the chordae tendineae do not play a significant role during mitral valve opening and do not influence the diastolic fluid dynamics^66^. A moving wall boundary condition was applied at the boundary surfaces between the fluid and solid inside the LV^65,66^. During diastole, there is an inflow from the atrium to the LV, but there is no outflow from the LV due to the closed aortic valve. Since the blood is incompressible^39^, interactions between the solid and fluid domains should be considered to conserve mass by allowing the blood to expand and contract the LV wall. The no-slip boundary condition was applied to the fluid–solid interface. In order to solve the FSI problem inside the nonlinearly deforming LV, we used the Robin boundary condition for pressure based on the approach proposed by Tukovic et al.^80^. The boundary condition for pressure was obtained from the following momentum equation ^80^:

25 $$\frac{\partial V}{{\partial t}} + (V - V_{s} ) \cdot \nabla V = \nabla \cdot (v\nabla V) - \frac{1}{\rho }\nabla p$$

At the non-permeable moving LV wall, the following equation holds^80^:

26 $$n \cdot \nabla p = - \rho \frac{{\partial V_{n} }}{\partial t}$$

where *V*_*n*_ is the normal component of LV wall and fluid velocity.

Fluid pressure at the interface was estimated by solid inertia as follows:

27 $$p \approx \rho_{s} h_{s} \frac{{\partial V_{n} }}{\partial t}$$

where *ρ*_*s*_ is the density of the LV structure and *h*_*s*_ is the LV thickness calculated as^81^:

28 $$h_{s} = \Delta t\sqrt {\frac{\lambda + 2\mu }{{\rho_{s} }}}$$

where *λ* and *µ* are Lame constants of the LV and *Δt* is the time step size. Finally, combining Eqs. (26) and (27) gives the Robin boundary condition for pressure^80^:

29 $$p + \frac{{\rho_{s} h_{s} }}{\rho }\frac{\partial p}{{\partial n}} = 0$$

Therefore, the coupled FSI problem employed the Robin-Neumann approach in which the fluid component used the Robin boundary condition for pressure, and subsequently, velocity was calculated based on that pressure.

#### FSI solution and strategy

Our FSI simulations relied on FOAM-Extend^37^ in which the system of equations governing the FSI problem were formulated using the finite volume method (See Fig. 2 for FSI algorithm flowchart). The system of equations governing the FSI problem was solved using a cell-centered finite volume method which is frequently used in CFD and is being increasingly used for solid modeling as well^82^.

The fluid model was discretized in space using the second-order accurate cell-centered finite volume method. Numerical integration in time was performed using the second-order backward Euler scheme. The coupling between pressure and velocity was performed using the segregated PISO algorithm^79,83^. The system of discretized equations was solved by a preconditioned Bi-Conjugate Gradient method^84^.

In the solid model, the second-order derivative was discretized using a second-order accurate backward scheme, proven to stabilize the numerical model^85^, to unify the discretization of the temporal terms between the fluid and solid discretization methods. The system of discretized equations was solved by a linear solver using a preconditioned Conjugate Gradient method^76^.

The moving boundary (interface) of the LV was controlled using a dynamic mesh methodology which was updated with the movement of the solid boundary. This method, based on the Laplace equation, was used for updating the computational and geometric nodes of the fluid mesh and was discretized by the cell-centered finite-volume method^79^. The systems of discretized equations were solved by a geometric agglomerated algebraic multi-grid solver.

We used the interface Quasi-Newton-Implicit Jacobian Least-Squares (IQN-ILS)^86^ algorithm to couple the discretized governing equations of the fluid and solid domains. The IQN-ILS method has been compared with the monolithic method and other partitioned methods such as Aitken’s dynamic relaxation and has been proven to have better performance and stability^86,87^. In this partitioned approach, traction was calculated at the fluid side of the interface and was used as a traction boundary condition at the solid side of the interface.

The calculations of the 3-D flow fields were done on a discrete mesh and the data obtained from our calculations were discrete. Continuous contours for the 3-D flow fields were created in Paraview (an open-source visualization software) using linear interpolation.

## Results

### Validation: non-invasive image-based diagnostic framework versus clinical Doppler echocardiography data

#### Trans-mitral velocity

Figures 3A–F and 4A–F compare the peak trans-mitral velocities for patients No. 1 to 3 between those simulated by our computational framework and those measured by DE (Fig. 3: Pre-TAVR, Fig. 4: Post-TAVR). There was a strong correlation between the simulated peak velocities and the ones measured by DE in all three patients, with a maximum relative error of: Pre-TAVR: 9.7% for E-waves and 8% for A-waves; Post-TAVR: 8.3% for E-waves and 8% for A-waves.

#### Left ventricle flow (apical view)

Depending on the direction of the flow, there are positive and negative values for DE velocity: red and blue colors represent blood flow towards and away from the transducer, respectively. As shown in Figs. 3G–I and 4G–I, both the magnitude and direction of flow demonstrate good qualitative and quantitative agreements between our computational results and the DE measurements.

#### Mitral valve flow (parasternal short axis & apical views)

Figures 3J–L and 4J–L investigated mitral valve inflow measured by DE and calculated with our computational framework (4 K: parasternal short axis; 3 J, 3 K, 3L, 4 J and 4L: apical view). Our results show good agreements between the velocities calculated using the computational framework and the ones measured using DE.

### Assessment of hemodynamics using current clinical routines

**Figure 5:**
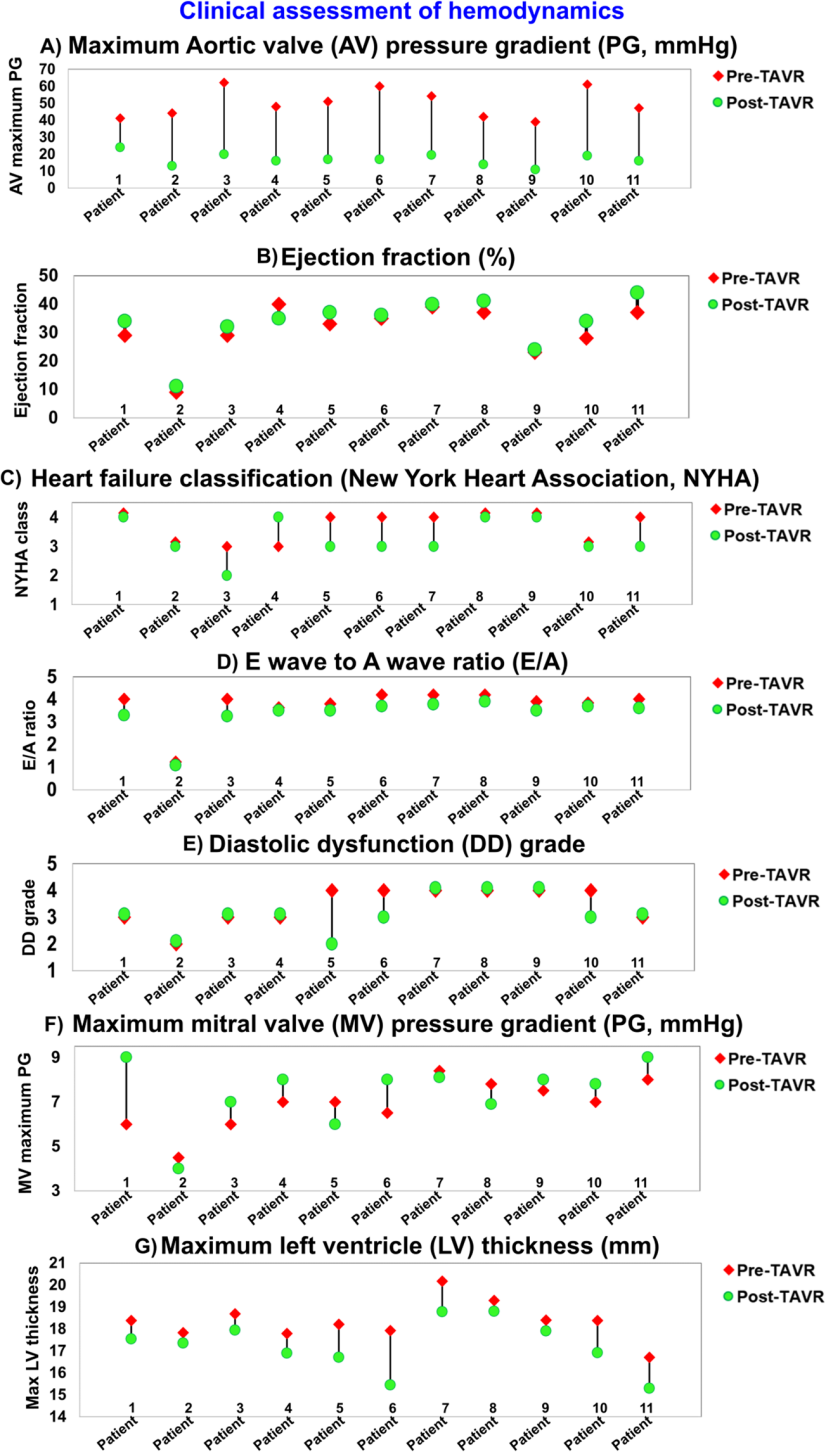
Changes in clinical assessment of hemodynamics in patients between baseline and 90-day post-TAVR (N = 11). (**a**) Maximum aortic valve pressure gradient; (**b**) Ejection fraction; (**c**) Heart failure classification; (**d**) E wave to A wave ratio (E/A); (**e**) Diastolic dysfunction grade; (**f**) Maximum mitral valve pressure gradient; (**g**) Maximum left ventricle thickness.

Changes of ventricular and valvular hemodynamic indices from baseline (prior to TAVR) to 90 days after TAVR are presented in Fig. 5. All patients who received TAVR were diagnosed with moderate to severe aortic stenosis^88^ (Severe AS is diagnosed based on a maximum aortic valve jet velocity > 4 m/s and a mean pressure gradient > 40 mmHg ^88^). Clinical assessment of AS for intervention decision-making was performed based on the symptoms of aortic valve hemodynamic metrics. As expected, aortic valvular metrics improved significantly after TAVR by removing the aortic valve obstruction that was causing an excessive pressure gradient and LV afterload. Our DE data showed that for all patients, TAVR significantly reduced the maximum pressure gradient across the aortic valve to a normal range^88^. Indeed, the maximum velocity measured less than 2.5 m/s and the maximum pressure gradient measured less than 25 mmHg for all patients after TAVR. The aortic valve maximum pressure gradient reductions ranged between 42% (patient #1) and 67% (patient #6) (Fig. 5A).

As an indicator of LV contractility, the ejection fraction (EF = (EDV-ESV)/EDV; EDV and ESV are end diastolic volume and end systolic volume, respectively) is considered to be abnormal once it is less than 41%^89^. Although the EF slightly increased for some patients after TAVR, with six patients demonstrating a 5–7% increase in EF, the EF still remained less than 41% for all patients from baseline to 90 days after intervention (Fig. 5B).

As an indicator of heart failure symptoms, the New York Heart Association (NYHA) functional class was determined for each patient based on the level of limitation in their ordinary activities, from 1 (no limitation in daily activity) to 4 (severe limitations in daily activity). All the patients had symptoms of heart failure, defined as a NYHA functional class greater than 2, both at baseline and at 90 days after TAVR. Post-TAVR, five patients’ symptoms slightly improved (from NYHA 4 to NYHA 3), five patients remained unchanged and one patient’s symptoms worsened (from NYHA 3 to NYHA 4) (Fig. 5C).

Diastolic dysfunction refers to impaired LV relaxation with or without an increase in filling pressure^25^. The diastolic dysfunction grade was obtained according to the American Society of Echocardiography recommendations based on the mitral valve velocity ratio of early diastolic velocity (E) to atrial contraction velocity (A), defined as the E/A ratio, as well as the annulus velocity (e^’^) as an index of LV diastolic filling efficiency^25^ (Fig. 5D). Diastolic dysfunction can be graded from 1 to 4; e.g. 1: Impaired relaxation, 2: Pseudo normal, 3: Reversible restrictive and 4: Fixed restrictive^90^. All patients had a diastolic dysfunction grade > 2 both at baseline and at 90 days after TAVR, meaning that they all had impaired filling at baseline, and no improvement was observed after TAVR (Fig. 5E). While an E/A ratio of 0.8 to 2 is considered to be normal^25^, most patients (85%) had an E/A > 2 both at baseline and at 90 days after TAVR; only one patient had E/A = 1.1 for both pre and post-TAVR. While the unchanged diastolic dysfunction gradient correlated with the E/A gradient before and after TAVR, these results did not correlate with the maximum mitral valve pressure gradient (Fig. 5F).

Maximum left ventricle thickness could be a potential indicator of mortality for patients with left ventricular hypertrophy (thickness > 15 mm)^91–93^. Although a moderate reduction in the maximum LV wall thickness was observed for all patients after TAVR (between 2.6% (patient # 2) and 14% (patient # 6)), the maximum LV thickness remained greater than 15 mm for all patients even after TAVR (Fig. 5G).

### Non-invasive image-based diagnostic framework: computed global hemodynamics (metrics of circulatory function and metrics of cardiac function)

#### Circulatory function

**Figure 6:**
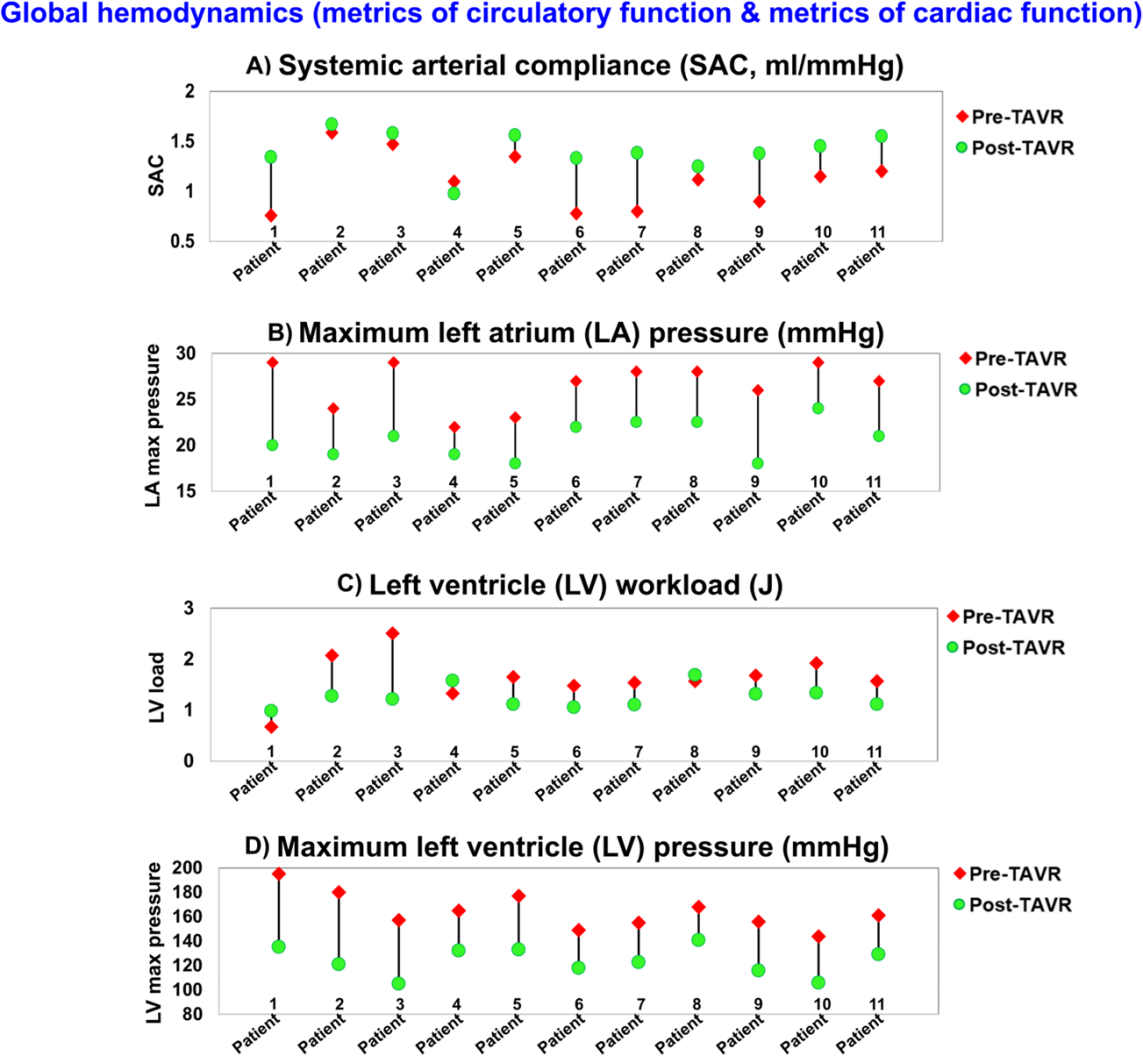
Changes in global hemodynamics (metrics of circulatory function & metrics of cardiac function) in patients between baseline and 90-day post-TAVR (N = 11). (**a**) Systemic arterial compliance; (**b**) Maximum left atrium pressure; (**c**) Left ventricle workload; (**d**) Maximum left ventricle pressure. *Global hemodynamics*: (1) Metrics of circulatory function, e.g., detailed information of the dynamics of the circulatory system, and (2) Metrics of cardiac function, e.g., heart workload and the breakdown of workload contributions from each cardiovascular disease component.

Systemic arterial compliance was obtained as an index of arterial hemodynamics. A low SAC (lower than 0.64 ml/m^2^/mmHg) is associated with an increased risk of morbidity for patients with AS^94^. As shown in Fig. 6A, SAC improved for the majority of patients after TAVR, with SAC increasing to > 1 (ml/mmHg) for all patients after intervention. For seven patients, the SAC increased between 16% (patient #5) and 77% (patient #1), but for the other four patients, SAC variations were less than 10%. The increase in SAC was associated with a decrease in maximum LA pressure for all patients as shown in Fig. 6B (from 14% (patient #4) to 31% (patient #1)). However, the maximum LA pressure was still greater than 18 mmHg for all patients even after TAVR (compared with normal LA pressure defined as < 15 mmHg ^95^). The increased LA pressure was correlated with high velocity during the E wave and a high E/A ratio during diastole (Fig. 5D) for both pre-TAVR and post-TAVR cases. Our results showed that the maximum LA pressure was universally reduced once the SAC increased after intervention. However, no improvement was observed in the E/A ratio (Fig. 5D) or in diastolic dysfunction grade (Fig. 5E).

#### Cardiac function

The LV workload is an effective metric of the LV load and clinical state^7,27^, and represents the energy that the ventricle delivers to the blood during ejection plus the energy necessary to overcome the viscoelastic properties of the myocardium itself. The LV workload is the integral of LV pressure and its volume change and was calculated as the area encompassed by the LV pressure–volume loop. Despite the universal reduction in the transvalvular pressure gradient, the LV workload did not improve (decrease) in all patients: TAVR reduced the LV workload in 70% of patients, but increased the workload in 30% of patients (Fig. 6C). Moreover, transvalvular pressure gradient reductions caused by TAVR did not always lead to an improvement in ejection fraction (Fig. 5B) or heart failure symptoms as measured by the NYHA score (Fig. 5C). Although TAVR did reduce the maximum LV pressure that was previously elevated by the increased LV burden from AS for all patients (Fig. 6D), no improvement was observed in diastolic dysfunction grade for 73% of the patients (Fig. 5E).

### Non-invasive image-based diagnostic framework: computed local hemodynamics (cardiac fluid dynamics)

The flow vortical structure inside the LV depends on the atrioventricular pressure, LV geometry, LV wall stiffness and mitral valve geometry. LV dynamics during diastolic filling, in particular, could play a crucial role in overall cardiac health^96^.

**Figure 7:**
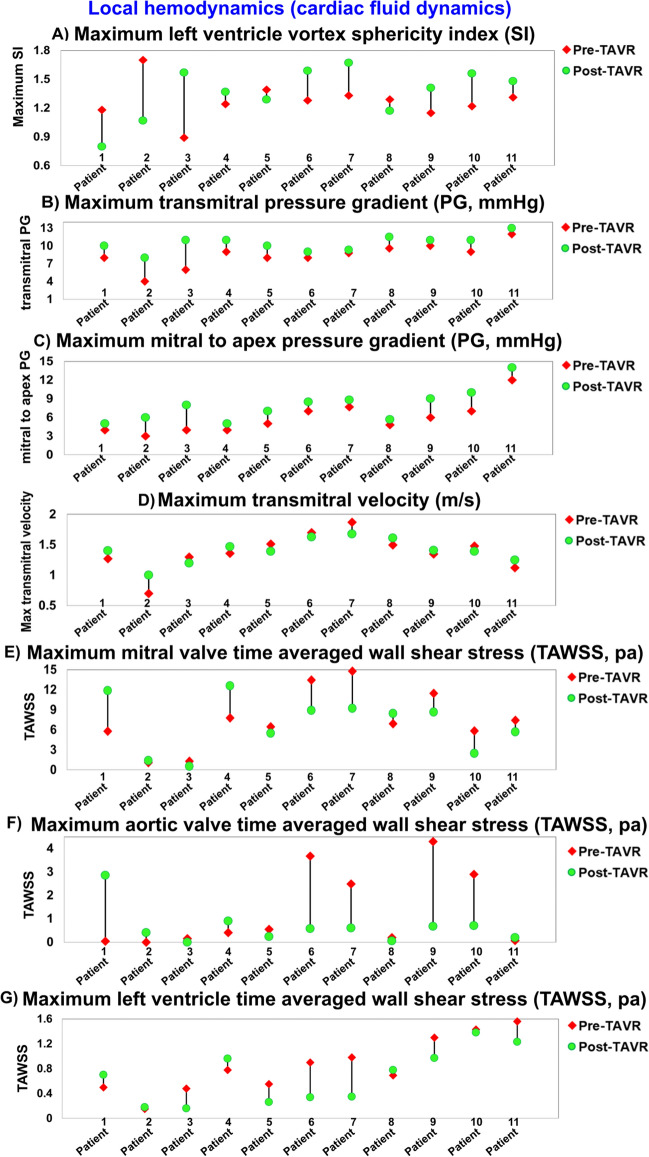
Changes in local hemodynamics (cardiac fluid dynamics) in patients between baseline and 90-day post-TAVR (N = 11). (**a**) Maximum left ventrcile vortex sphericity index; (**b**) Maximum transmitral pressure gradient; (**c**) Maximum mitral to apex pressure gradient; (**d**) Maximum transmitral velocity; (**e**) Maximum mitral valve TAWSS; (**f**) Maximum aortic valve TAWSS; (**g**) Maximum left ventricle TAWSS. *Local hemodynamics*: cardiac fluid dynamics, e.g., details of the instantaneous 3-D flow and vortex formation.

**Figure 8:**
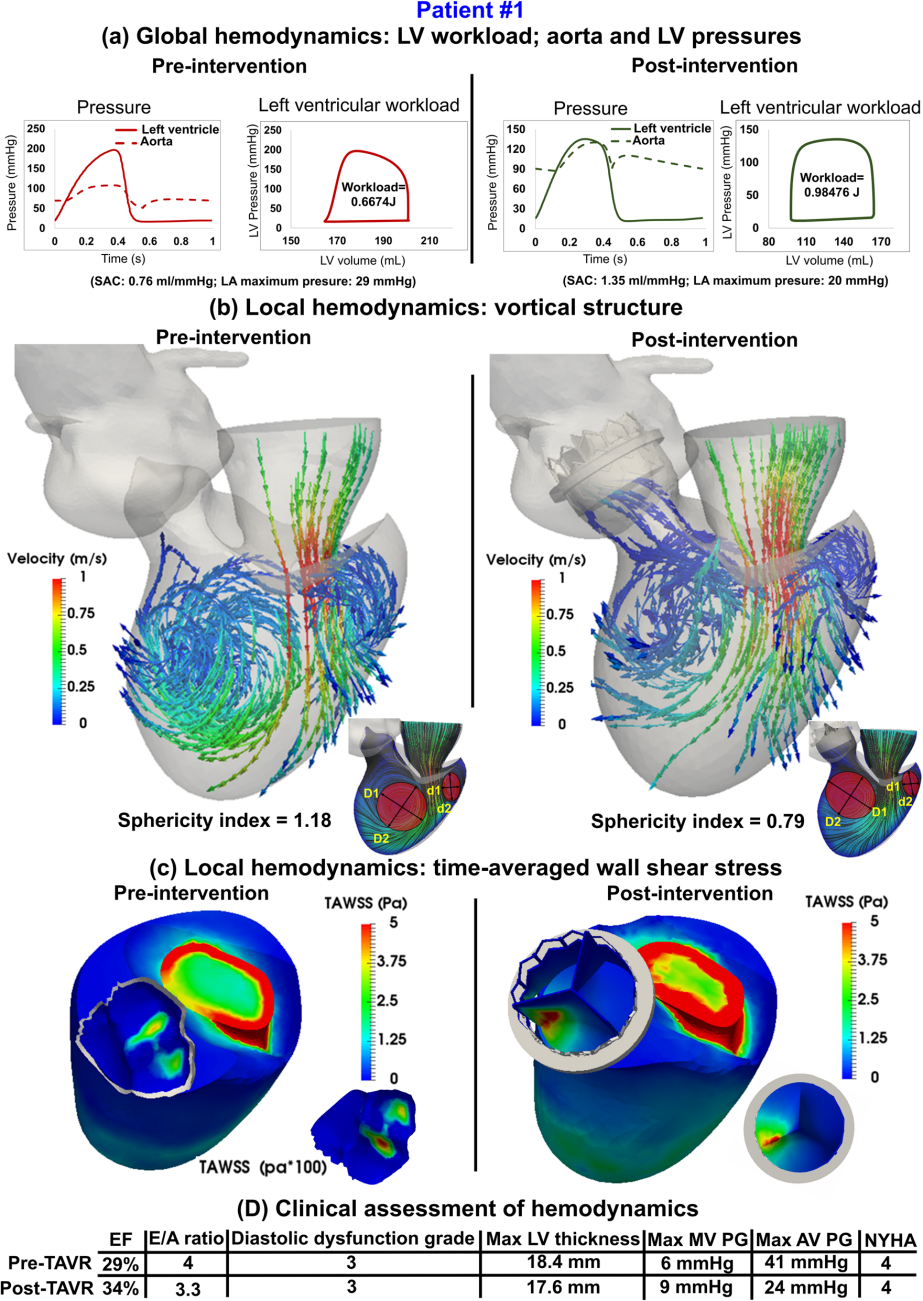
Changes in local and global hemodynamics in patient #1 between baseline and 90-day post-TAVR. (**a**) *Global hemodynamics*: LV workload, aorta and LV pressures; (**b**) *Local hemodynamics*: vortical structure; (**c**) *Local hemodynamics*: time-averaged wall shear stress; (**d**) Clinical assessment of hemodynamics. Local hemodynamics: *cardiac fluid dynamics*, e.g., details of the instantaneous 3-D flow and vortex formation. Global hemodynamics: (1) *Metrics of circulatory function*, e.g., detailed information of the dynamics of the circulatory system, and (2) *Metrics of cardiac function*, e.g., heart workload and the breakdown of workload contributions from each cardiovascular disease component.

**Figure 9:**
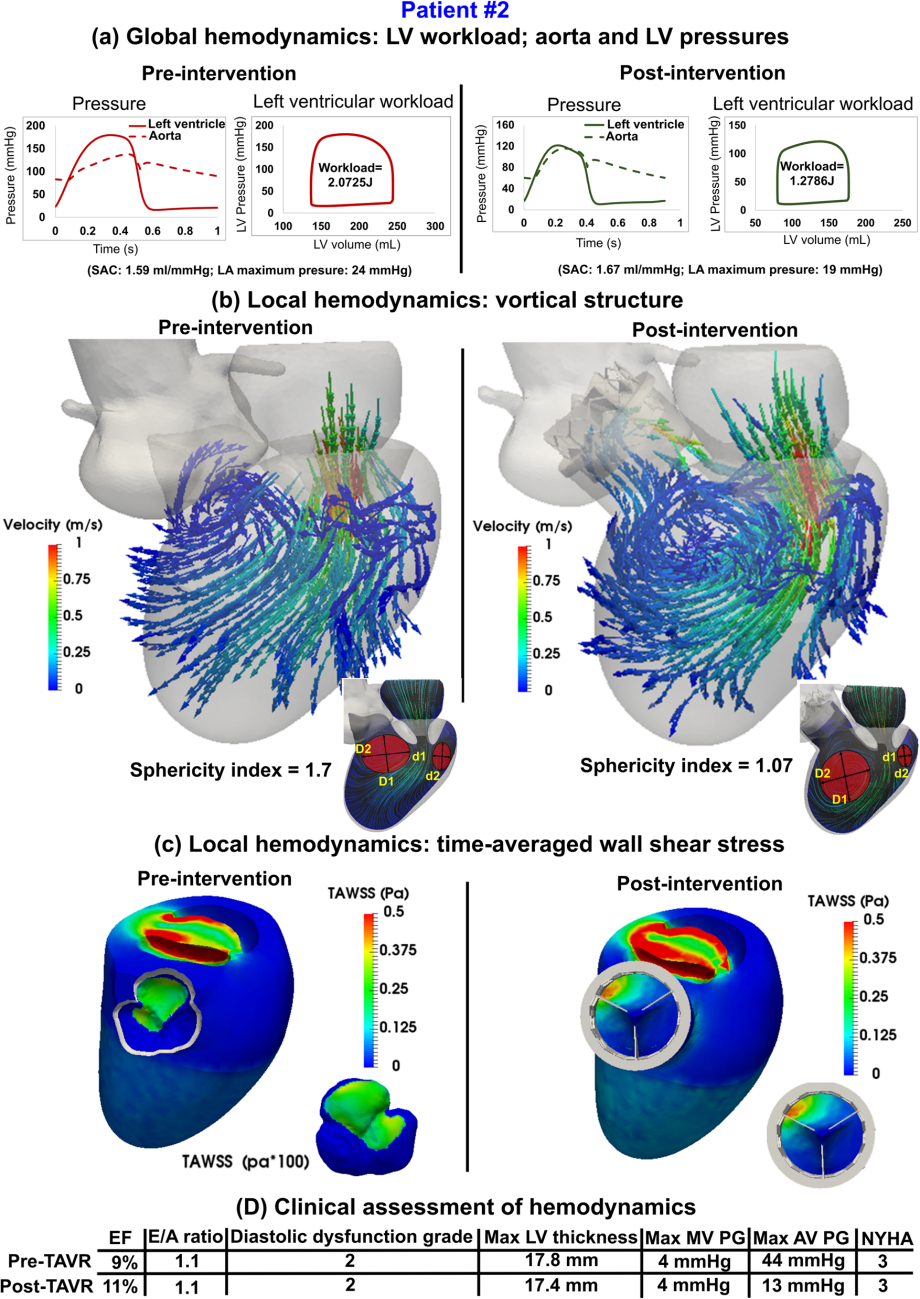
Changes in local and global hemodynamics in patient #2 between baseline and 90-day post-TAVR. (**a**) *Global hemodynamics*: LV workload, aorta and LV pressures; (**b**) *Local hemodynamics*: vortical structure; (**c**) *Local hemodynamics*: time-averaged wall shear stress; (**d**) Clinical assessment of hemodynamics. Local hemodynamics: *cardiac fluid dynamics*, e.g., details of the instantaneous 3-D flow and vortex formation. Global hemodynamics: (1) *Metrics of circulatory function*, e.g., detailed information of the dynamics of the circulatory system, and (2) *Metrics of cardiac function*, e.g., heart workload and the breakdown of workload contributions from each cardiovascular disease component.

**Figure 10:**
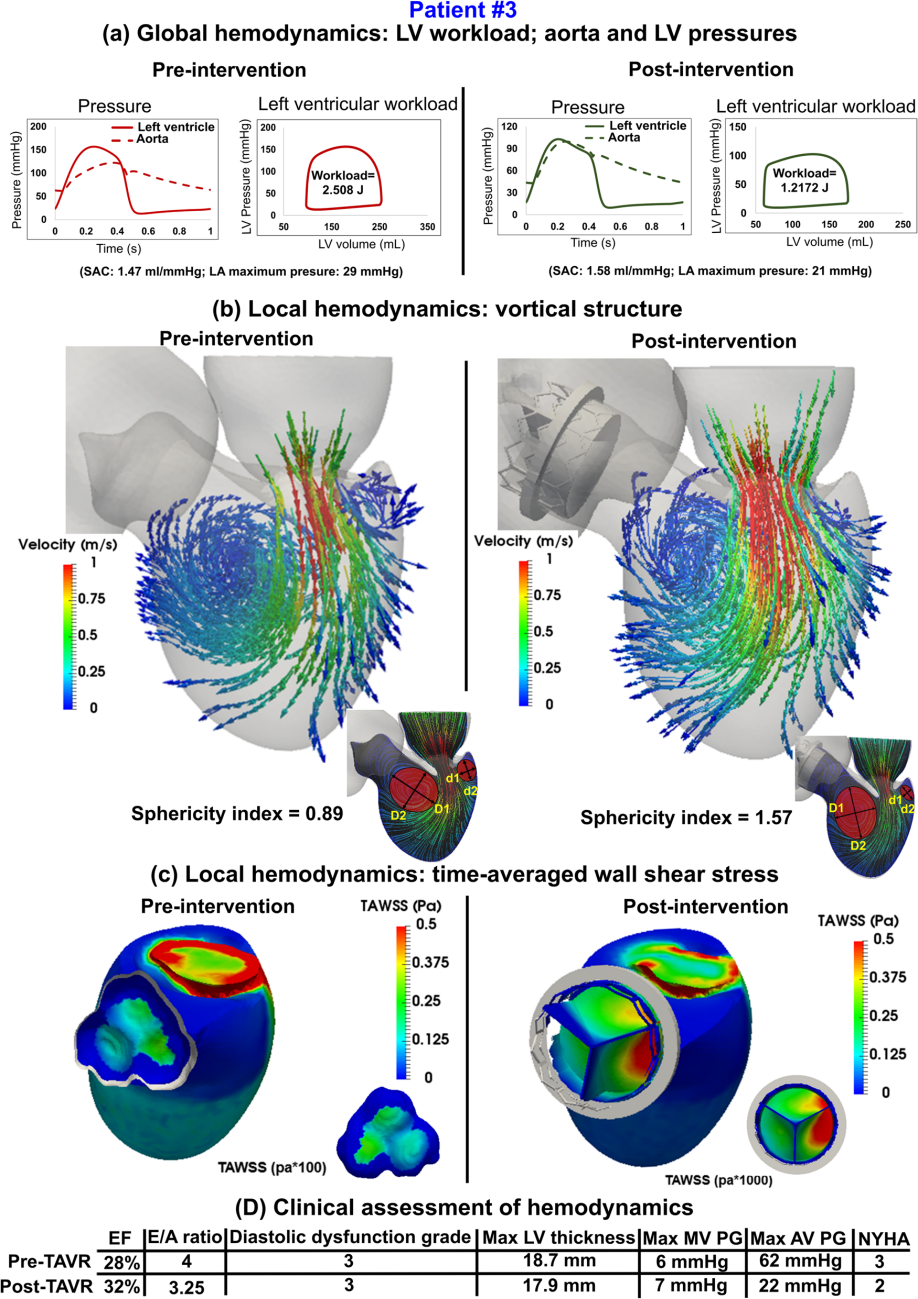
Changes in local and global hemodynamics in patient #3 between baseline and 90-day post-TAVR. (**a**) *Global hemodynamics*: LV workload, aorta and LV pressures; (**b**) *Local hemodynamics*: vortical structure; (**c**) *Local hemodynamics*: time-averaged wall shear stress; (**d**) Clinical assessment of hemodynamics. Local hemodynamics: *cardiac fluid dynamics*, e.g., details of the instantaneous 3-D flow and vortex formation. Global hemodynamics: (1) *Metrics of circulatory function*, e.g., detailed information of the dynamics of the circulatory system, and (2) *Metrics of cardiac function*, e.g., heart workload and the breakdown of workload contributions from each cardiovascular disease component.

In order to explain the vortex morphology more precisely, the vortex sphericity index was calculated in the long axis view (2-D plane) by dividing the vortex length (D2) by the vortex width (D_1_) (Figs. 7A, 8B, 9B and 10B)^97,98^. A normal vortex sphericity index (SI) is defined to be greater than 2, and a lower SI is associated with a higher risk of apex thrombosis or myocardial infarction^98^. For all patients in our study, the vortex sphericity index was lower than 2 both at baseline and 90 days after TAVR (maximum SI = 1.7), making the vortex more spherical and thus increasing the risk of thrombosis at the apex (Fig. 7A). While 2/3 of patients experienced a slight increase in SI with improved filling function after TAVR, the rest (1/3 of patients) had a decrease in SI and a worsened filling pattern. Since the SI depends on upstream flow, metrics of circulatory and cardiac function, geometrical details of the mitral valve and LV, and LV relaxation, the SI can either be improved or worsened depending on the interactions between these parameters. It is crucial to prevent the reduction of the SI following TAVR because this could lead to thrombus formation and adverse outcomes in the flow transferring mechanism from the atrium towards the left ventricular outflow tract^97,98^. This can be explained by the fact that when the elongated shape of vortex turns into a more circular shape, the apex of the LV is not exposed to a fast-moving blood flow, and thus, the flow separated from the vortex in the main stream could lead to the development of a thrombus (more prone at the apex or septum). This is an established complication in many cardiac conditions, with the highest rate detected in myocardial infarction and congestive heart failure.

As previously mentioned, our results showed that pressure reduced after TAVR both for the left ventricle and the left atrium (Fig. 6, global hemodynamics). However, this does not always lead to an improved pressure gradient (PG) between the atrium and left ventricle after TAVR. Indeed, the maximum transmitral PG did not change for 36% of the patients, slightly increased for 45% of the patients and considerably increased for only 19% of the patients (Fig. 7B). We also computed the PG from the atrium to apex to compare with the transmitral gradient. Although this diagram shares the same general trend as the transmitral PG, overall, the mitral to apex PG is lower than the transmitral PG (Fig. 7C). Interestingly, this difference between the PGs (Fig. 7B and C) is correlated with more spherical vortices (SI < 2) behind the anterior leaflet of the mitral valve, which causes less flow to be driven towards the apex and septum. In other words, a more spherical vortex leads to a reduced mitral-to-apex PG when compared to the transmitral PG. Moreover, no significant difference was observed in the maximum transmitral velocity (Fig. 7D) for majority of the patients (82%), which is in agreement with the results of maximum transmitral PG (Fig. 7B).

Wall shear stress, as a force induced by blood flow, has a major impact on regulating endothelial function^99^. In general, very high wall shear stress could damage the tissue and very low wall shear stress could lead to thrombus formation^99^. It has been reported that wall shear stress for a normal LV lies between 0.2 and 1.2 Pa^65,100^ and increases slightly as a result of hypertrophy^100^. The total shear stress exerted on the wall throughout the cardiac cycle was evaluated using the time-averaged wall shear stress (TAWSS) which is obtained as ${\text{TAWSS}} = \frac{1}{{\text{T}}}\mathop \smallint \limits_{0}^{{\text{T}}} \left| {\uptau } \right|{\text{dt}}$ (T and ${\uptau }$ are the cardiac cycle period and instantaneous wall shear stress, respectively). We calculated TAWSS during diastole for all cases in both pre and post TAVR states. The maximum local TAWSS was increased at both the mitral valve and aortic valve leaflets for 27% of the patients and at the LV for 20% of the patients (Fig. 7E–G)—Such high TAWSS could be a concern for patients who received TAVR. On the other hand, our findings showed that the aortic valve and LV could be at risk of thrombus formation for some patients as a result of very low TAWSS after TAVR (that is associated with a low velocity region around the ventricular side of the leaflets); the maximum TAWSS decreased significantly for 37% of the patients at the aortic valve (Fig. 7F) and for 55% of patients at the LV (Fig. 7G).

## Discussion

*Quantification of the complex flow plays an essential role in accurate and early diagnosis of patients with C3VD*^101,102^. It can be used to effectively plan interventions and make critical clinical decisions with life-threatening risks*.* Once a C3VD patient develops symptoms, intervention becomes a class I recommendation^103,104^. However, at the time of diagnosis, symptoms are not reported by almost 40% of these patients^105^. Furthermore, there are often discrepancies found during clinical evaluation in one third of C3VD patients^105,106^. It is therefore essential to accurately diagnose individuals through careful hemodynamic evaluation to identify who may benefit from intervention (e.g., TAVR) and experience improved outcomes^106–108^. Overall, upon diagnosis, the results of anatomic and hemodynamic measures should agree^107^. Thus, accurate quantification of hemodynamic parameters is critical to resolve any inconsistencies and to identify the optimal course of treatment for each individual patient^106,107^. With an increasing appreciation for the hypothesis that valvular disease is complex and is also influenced by the principals of the LV and arterial system, quantitative investigations of hemodynamics that consider the interactive coupling of the valve, ventricle and arterial system have become extremely desirable^109–112^. The following three requirements should be quantified by a clinically useful computational diagnostic framework that evaluates both local and global hemodynamics for patients with C3VD and TAVR:

(1) *Metrics of circulatory function (global hemodynamics).* The heart resides in a sophisticated vascular network whose loads impose boundary conditions on the heart function^7,27,33,102,113^. Furthermore, it is critical to replicate the correct flow and pressure conditions when developing a patient-specific cardiovascular simulator because the local flow dynamics are influenced by both downstream and upstream conditions. This ensures that patient-specific flow and pressure conditions are provided to the local flow while also enabling analysis of the effects of local hemodynamics on the global circulatory physiology. Complex valvular, ventricular and vascular diseases (C3VD) is the most fundamentally challenging cardiovascular pathology, in which several pathologies have mechanical interactions with one another wherein adverse physical phenomena associated with each pathology amplify the effects of others on the cardiovascular system^4–9,27^. TAVR often coexists with C3VD, thus making the investigation of flow and pressure details in the presence of TAVR very challenging. Although *cardiac catheterization* is currently the clinical gold standard for evaluating the global function of the heart and circulatory system using pressure and flow measurements, it is not practical for diagnosis in routine daily clinical practice or serial follow-up examinations, as it is invasive, expensive and high risk^14^. Furthermore, it is important to note that cardiac catheterization does not provide details of the physiological pulsatile flow and pressures throughout the heart and circulatory system, but instead, only enables access to blood pressure in very limited regions.

Effective diagnosis is critically dependent on quantifying details of the physiological pulsatile flow and pressures throughout the heart and circulatory system as well as the interactions within C3VD and how individual disease progressions may affect one another^114–120^. Indeed, due to these interactions, several regions throughout the heart and surrounding system are often affected ^118–120^, and certain conditions of the circulatory system may prevent the accurate assessment of C3VD or affect the outcomes of TAVR^106^. For example, from a large registry of C3VD patients undergoing endovascular TAVR, almost one quarter suffered from coexisting peripheral artery disease, which was found to be associated with higher odds of vascular complications and major bleeding^121^. Moreover, regardless of the flow conditions, the presence of hypertension or reduced arterial compliance in patients with C3VD may reduce the transvalvular gradient and peak transvalvular velocity, thus causing an underestimation of aortic stenosis severity^106^. Hypertension is also a risk factor for cardiovascular events and may be associated with worse outcomes and faster progression of C3VD^106,116^. Overall, precise knowledge of these interactions and careful assessment of hemodynamics in a patient-specific manner helps optimize the diagnosis process to provide the best possible outcomes for patients^107,120^, to decide upon the required course of treatment and determine if more than one intervention is required for the C3VD patients^106,117^.

(2) *Metrics of cardiac function (global hemodynamics)*. In the presence of TAVR and/or C3VD, the heart is overloaded since the healthy instantaneous left-ventricle pressure and/or left-ventricle flow are altered^7,27^. In clinics, *cardiac catheterization* is the gold standard for evaluating heart function in terms of the heart workload obtained from the instantaneous left-ventricle pressure and/or left-ventricle flow. However, *there is no method to invasively or non-invasively quantify the heart workload (global function)* that can provide the contribution breakdown of each component of the cardiovascular system. This is especially crucial in C3VD and TAVR because quantification of the left-ventricle workload and its breakdown are vital to guide the prioritization of interventions and to sufficiently validate devices in regulatory testing machines. Moreover, there *is no non-invasive method for determining left-ventricular end-diastolic pressure, instantaneous left-ventricular pressure, and contractility*—all of which provide valuable information about the patient’s state of cardiac deterioration and heart recovery.

In patients with C3VD, the valves and left ventricle are diseased, thus altering the overall cardiac function^122,123^. Following TAVR, in many cases, there is improvement in the structure and function of the left ventricle, with regression of the myocardial cellular hypertrophy and diffuse fibrosis^122^. However, the development of focal fibrosis, which provides evidence of cardiomyocyte necrosis, is irreversible^122^. To optimize the intervention outcome before the ventricle is permanently damaged^114,124^, to choose the optimal time of intervention^115,125^, and to drastically reduce the risk of mortality^115^, knowledge of the heart workload (cardiac function) and the contribution breakdown of each component in C3VD should be precisely quantified and evaluated at the time of diagnosis.

(3) *Cardiac fluid dynamics (local hemodynamics)*. The complex pulsatile flow in the left ventricle and its valves becomes even more complicated in C3VD. Chirality of the human heart causes this flow to be strongly three dimensional^10,96^. Moreover, as a result of TAVR, new interactions occur between the artificial implant and the native valve geometry, thus altering the fluid dynamics^126^. During filling of the normal heart, the blood entering the left ventricle through the mitral valve forms a vortex that minimizes energy dissipation, prevents blood stagnation and optimizes pumping efficiency^10,96^. C3VD and TAVR alter this optimized flow^7^: the vortex dynamics become less synchronized with the heart contraction, and vortices other than the healthy vortex ring may emerge and interact with one another. To predict the success of TAVR and plan the best deployment possible in each patient, it is crucial to know details of the instantaneous 3-D flow, vortex formation, growth, eventual shedding, and their effects on fluid transport and stirring inside the left ventricle and in the vicinity of the valves after deployment^10,96,127^. It is essential for a diagnostic tool to carefully quantify and predict cardiac fluid dynamics in a patient-specific manner because there can be high inter-patient variability in the success of any given intervention^128^. Altered hemodynamics should be optimized with treatment, as they can lead to adverse outcomes such as an increased risk of thrombus formation^126^. One meta-analysis identified the risk of stroke being four times greater in TAVR patients with leaflet thrombosis^129^.

A clinically-useful computational diagnostic framework should quantify both local and global hemodynamics. As examples: (1) Patient #1 (Fig. 8); global hemodynamics: Circulatory function improved after TAVR; SAC increased from 0.76 ml/mmHg to 1.35 ml/mmHg and maximum atrium pressure decreased from 29 to 20 mmHg. However, circulatory function improvements were not associated with improvement of cardiac function. LV workload was adversely increased due to the paravalvular leakage after TAVR (Fig. 8A: the workload increased from 0.67 J to 0.98 J). local hemodynamics: the increased workload led to early breakdown of the flow as a result of the interaction of paravalvular leakage with the mitral valve (Fig. 8B); maximum SI decreased from 1.18 to 0.79, leading to the formation of a less intense spherical vortex behind the anterior leaflet that depicts worsening of the diastolic flow pattern after TAVR. Moreover, the maximum TAWSS increased significantly after TAVR, from 0.04 Pa to 3 Pa for the aortic valve leaflets and from 5.77 Pa to 11.86 Pa for the mitral valve leaflets. Such a significant increase in TAWSS resulted from a disturbed flow pattern around both valves. The spatial shift of the affected location following TAVR provides another critical factor while localizing the maximum TAWSS: the maximum TAWSS on the aortic valve shifted from the posterior and left coronary cusps to the right and left coronary cusps (Fig. 8C). In summary, TAVR removed the aortic valve obstruction, reduced aortic valve pressure gradient and increased the ejection fraction in patient #1. However, considering cardiac function and local flow variations, this patient is at a high risk of heart failure and did not fully benefit from TAVR; (2) Patient #2 (Fig. 9); global hemodynamics: both the circulatory and cardiac functions improved after TAVR; SAC increased from 1.59 ml/mmHg to 1.67 ml/mmHg, maximum atrium pressure decreased from 24 to 19 mmHg and LV workload decreased from 2.07 J to 1.28 J (Fig. 9A). local hemodynamics: the improvements of global hemodynamics were associated with an improved vortical structure; the reduced workload after TAVR was associated with slightly improved LV relaxation, which let the vortex moves forward and becomes closer to the apex prior to its interaction with the LV wall and its subsequent dissipation. Although the SI decreased for this patient, the isolated pre-TAVR vortex, located behind the anterior leaflet, shifted towards the center of the LV after TAVR (Fig. 9B). This spatial shift of the vortex center facilitated the filling mechanism. Moreover, vortex alterations after TAVR led to a considerable increase of TAWSS at the aortic valve leaflets from 0.0015 Pa (with a high risk of thrombosis) to 0.4 Pa. TAVR improved the overall global and local hemodynamics, although no changes were observed in clinical hemodynamic assessment of diastolic function after TAVR (such as E/A ratio, max mitral valve PG or EF); (3) Patient #3 (Fig. 10); global hemodynamics: both the circulatory and cardiac functions improved after TAVR; SAC increased from 1.47 ml/mmHg to 1.58 ml/mmHg, maximum atrium pressure decreased from 29 to 21 mmHg and LV workload decreased from 2.508 J to 1.22 J (Fig. 10A). local hemodynamics: this global improvement of hemodynamics was associated with a vortex that was more elongated towards the apex with improved filling efficiency; the maximum SI increased from 0.89 to 1.57 after TAVR (Fig. 10B). However, the maximum TAWSS at the aortic valve reduced significantly from 0.145 Pa (at the center of the aortic leaflets ) to 0.00052 Pa (on the left coronary cusp) after TAVR (Fig. 10C), which is significantly lower than the minimum control value of 0.06 Pa^130^. Therefore, although clinical assessments have shown improvements (increased EF, decreased aortic valve pressure gradient and decreased NYHA classification), geometrical alterations at the left ventricular outflow tract after TAVR could increase the risk of thrombus formation. Indeed, a recent study showed that left ventricular outflow tract calcification increases the risk of annular rupture and residual aortic regurgitation^131^. Therefore, despite the improvements of clinical parameters, our results depicting the details of local hemodynamics in this patient might partially explain how TAVR could adversely increase the risk of LVOT calcification and subsequent long-term complications.

A clinically-useful computational diagnostic framework that can quantify both local and global hemodynamics for patients with C3VD and TAVR should quantify the three requirements mentioned in the Introduction and Discussions. Several studies have used computational fluid dynamics (CFD) based on the discretization of the Navier–Stokes equations (finite volume method, finite element method, etc.) with a moving boundary in an attempt to quantify blood flow (local hemodynamics) inside the LV, but none of these studies considered LV tissue thickness or other tissue characteristics^40,59,62,64,66,132–136^. In addition, several researchers have recently used FSI as a promising tool for computational cardiology because it allows for the complete coupling of the heart wall and blood flow mechanics, thus demonstrating its worth as the most comprehensive tool for numerical modeling of the LV^60,137–154^. However, since: (1) patient-specific boundary conditions were not used; (2) normal valves and ventricles were modeled instead of those with C3VD; and (3) patient-specific geometries were not used, the models developed in these studies didn’t satisfy the three requirements outlined in the Introduction^60,137–154^. While some models were partially validated using DE^145,152^ or MRI^60^, many were not validated. Five of the studies^60,74,137,143,147^ did impose boundary conditions on the calculations by coupling fluid–structure modeling calculations with lumped-parameter modeling, but the lumped-parameter models were either not patient-specific and/or they required information from MRI. MRI is not feasible in patients with implanted devices, and it is not available in all clinics, therefore restricting the collection of the necessary blood-flow and geometrical measurements. Additionally, idealized geometries were used in these studies, which could significantly affect the flow and vortex structure.

In this study, we developed an innovative computational diagnostic framework for complex diseases like C3VD and TAVR that dynamically couples the local hemodynamics (using a 3-D strongly-coupled fluid–solid interaction; FSI) with the global circulatory cardiovascular system (using the lumped-parameter algorithm) and satisfies the three requirements. This computational diagnostic framework is promising for future clinical adoption and can quantify: (1) metrics of circulatory function (global hemodynamics); (2) metrics of cardiac function (global hemodynamics) as well as (3) cardiac fluid dynamics (local hemodynamics) in patients with C3VD in both pre and post intervention states. Such information is vitally needed for effectively using advanced therapies to improve clinical outcomes and guide interventions in C3VD patients.

Due to the complex multiphysics nature of the left ventricle and heart valves, the overall estimation of cardiac parameters is very dependent on the outputs of the lumped-parameter model that are in-turn depend on the parameters used in the lumped-parameter model. Our patient-specific Doppler-based lumped-parameter algorithm, which provided boundary conditions, was validated against clinical catheterization data in forty-nine C3VD patients with a substantial inter- and intra-patient variability with a wide range of disease^24^. In the present study, we used the validated lumped-parameter model^24^ to obtain time varying pressure and volume of the left ventricle as the inputs to the solid model of the LV. We modeled the LV as an isotropic Saint Venant–Kirchhoff solid and found material parameters that could best reproduce the LV volume changes (obtained from the lumped-parameter model^24^ while applying LV pressure (obtained from the lumped-parameter model) to the LV wall. Using this approach, in all patients that we have investigated in this study, we could always find material parameters that produce consistent results with the lumped-parameter model. Moreover, we performed a comprehensive parameter sensitivity analysis on the outputs of the lumped-parameter model that are used in the present study to find cardiac parameters. We found that the outputs from the lumped-parameter model were most sensitive to the forward left ventricular outflow tract stroke volume (Forward LVOT-SV, an input parameter to the lumped parameter algorithm): LV pressure: 27%, LV Volume 19% by a ± 20% change in the Forward LVOT-SV. The other input parameters affected the output to a much lower degree. We should point out that Forward LVOT-SV is measured reliably using Doppler echocardiography with high accuracy and sensitivity of the model to this parameter does not jeopardize the results obtained from the model. In addition, sensitivity analysis revealed negligible effects of changes (± 20%) in the free parameters on the model output variables. Indeed, as shown in Figs. 3 and 4 in this study, the results obtained with fluid–structure interaction and lumped-parameter algorithm were validated against clinical Doppler echocardiography in patients. Our results show good agreements between velocity calculated using the computational framework and the ones measured using Doppler echocardiography in all investigated patients in both pre and post-TAVR intervention states.

## Limitations

This study was performed and validated on 11 patients with C3VD and TAVR using a 3-D strongly-coupled fluid–solid interaction and lumped-parameter modeling framework in both pre and post intervention states (22 cases). Future studies must consider further validation of the computational framework in a larger population of C3VD patients. However, our results in this study demonstrate the ability of the framework to track changes in both cardiac and vascular states. Our LPM algorithm allows analysis of any combination of complex valvular, vascular and ventricular diseases in both pre and post intervention conditions. It is important to note that this algorithm was validated against clinical catheterization data in forty-nine C3VD patients with a substantial inter- and intra-patient variability with a wide range of disease^24^. These observations made us more confident that the limitation in the number of patients in this study does not affect our conclusions.

## Data Availability

Upon acceptance all data will become available at https://dataverse.scholarsportal.info/.
